# Advances in Single‐Cell Sequencing for Infectious Diseases: Progress and Perspectives

**DOI:** 10.1002/advs.202415678

**Published:** 2025-07-04

**Authors:** Mengyuan Lyu, Yang Liu, Jian Zhou, Hongli Lai, Hongxia Ruan, Dongsheng Wu, Shun Zhu, Xudong Zhou, Wananqi Ma, Yuchen Huang, Shuting Lei, Han Luo, Jie Chen, Binwu Ying

**Affiliations:** ^1^ Department of Laboratory Medicine West China Hospital Sichuan University Chengdu Sichuan 610041 P. R. China; ^2^ Department of Thoracic Surgery West China Hospital Sichuan University Chengdu Sichuan 610041 P. R. China; ^3^ West China School of Medicine Sichuan University Chengdu Sichuan 610041 P. R. China; ^4^ Department of Division of Thyroid and Parathyroid Surgery West China Hospital Sichuan University Chengdu Sichuan 610041 P. R. China

**Keywords:** cell exhaustion, immunity, infectious diseases, interferon, single‐cell sequencing

## Abstract

Infectious diseases are a persistent threat throughout the twenty‐first century, owing to their widespread transmission mediated by global connectivity. Benefiting from the advancement of eukaryotic, prokaryotic (e.g., MATQ‐seq and BacDrop), and dual (e.g., PatH‐Cap) single‐cell sequencing technologies, changes in each side and host‐pathogen interactions can be elucidated. Pathogens undergo adaptive evolution in the host microenvironment, exhibiting different transcription profiles (e.g., upregulated HSV‐1 immediate‐early genes during infection), transcription efficiencies, and others. Consequently, they develop distinct replication capacity, virulence, and other phenotypes. For the host, intrinsic differences among cells and variations brought by diverse phenotypic pathogens together determine the heterogeneity of its responses (e.g., the dual roles of interferon‐I at different infection stages). Interferon‐related pathway alterations (influencing both adaptive and innate immune responses) and cell exhaustion (manifesting as the functional impairment of cells) are observed in various infections, seemingly as a shared approach in host‐pathogen interactions. These underlying commonalities provide a basis for the development of broad‐spectrum therapeutic targets. Collectively, this work reviewed recent progress of single‐cell sequencing technologies and their applications in infectious diseases, aiming to facilitate the development of powerful diagnostic or therapeutic strategies and thus advance precision medicine.

## Graphical abstract

Single‐cell sequencing technologies uncover novel, unknown, and emergent features of many diseases. This review describes recent progress of single‐cell sequencing technologies and their applications in infectious diseases, summarizes the underlying commonalities of different infections and discusses future research directions, facilitating the development of promising diagnostic or therapeutic strategies.

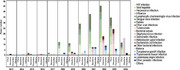

## 1 Introduction

Despite medical advancements and improved healthcare over the past two decades, infectious diseases caused by various pathogens remain formidable threats to public health, particularly in low and lower‐middle‐income countries. Human immunodeficiency virus (HIV) infection/acquired immune deficiency syndrome (AIDS), tuberculosis (TB), and malaria are contributing to high mortality and morbidity. The twenty‐first century has witnessed a succession of severe infectious disease outbreaks, with the coronavirus disease‐2019 (COVID‐19) pandemic being an unexpected event. These outbreaks of emerging, re‐emerging, and endemic pathogens, characterized by rapid and wide‐ranging spread, have caused extensive loss of human lives, destabilized economies, and adversely affected the overall welfare of societies.^[^ ^1^ ^]^ Deepening our understanding of infectious diseases is urgently needed.

It is well known that pathogens and hosts can reshape each other, which is manifested as genome integration, transcriptome alterations, epigenetic changes, and others. Decoding the driving factors and biological effects behind such changes is crucial for understanding the infection process of pathogens, host defense mechanisms, pathogen resistance, and so on. Traditional approaches to dissecting these diseases, mainly based on bulk‐level techniques, provide an average measurement of the above variations,^[^ ^2^, ^3^ ^]^ advancing the preliminary understanding of diseases. However, such average measurement masks some heterogeneity (e.g., rare cellular subclusters, transitional cell states with distinct molecular signatures), restricting the capture of host‐pathogen interaction complexity and individualization. For instance, some new cell clusters, including myeloid dendritic cells (DCs) characterized by CD64^Hi^ PD‐L1^Hi^, are found to have the potential to resist HIV,^[^ ^121^ ^]^ which may not have been discovered by bulk‐level techniques. While single‐cell sequencing technologies can offset the above limitation and allow researchers to capture the details of the reshaping process by enabling high‐resolution characterization of single cells. Nowadays, the complexity and spatiotemporal dynamics of infectious diseases have been gradually recognized, and the interpretability of heterogeneous disease phenotypes has been improved.^[^ ^4^, ^5^, ^6^ ^]^ Also, it has driven certain decisive changes, including the discovery of powerful biomarkers or targets, and the improvement of vaccine and immunotherapy strategies.^[^ ^7^, ^8^, ^9^ ^]^

Herein, this review first summarizes advancements in single‐cell sequencing technologies, including eukaryotic, prokaryotic, and dual single‐cell sequencing techniques. Subsequently, this work systematically synthesized applications of these technologies across various infectious diseases (prion, viral, bacterial, fungal, and parasitic infections). Finally, the commonalities of different host‐pathogen interactions were summarized, and future research directions were discussed. This review aims to offer theoretical evidence for single‐cell sequencing applications in infectious disease research, with the tripartite objectives of advancing clinical translation of this technology, facilitating the development of promising diagnostic biomarkers and therapeutic targets, and ultimately mitigating the global disease burden through precision medicine approaches.

## 2 Development of Single‐Cell Sequencing Technologies in Infectious Diseases

**Figure 1:**
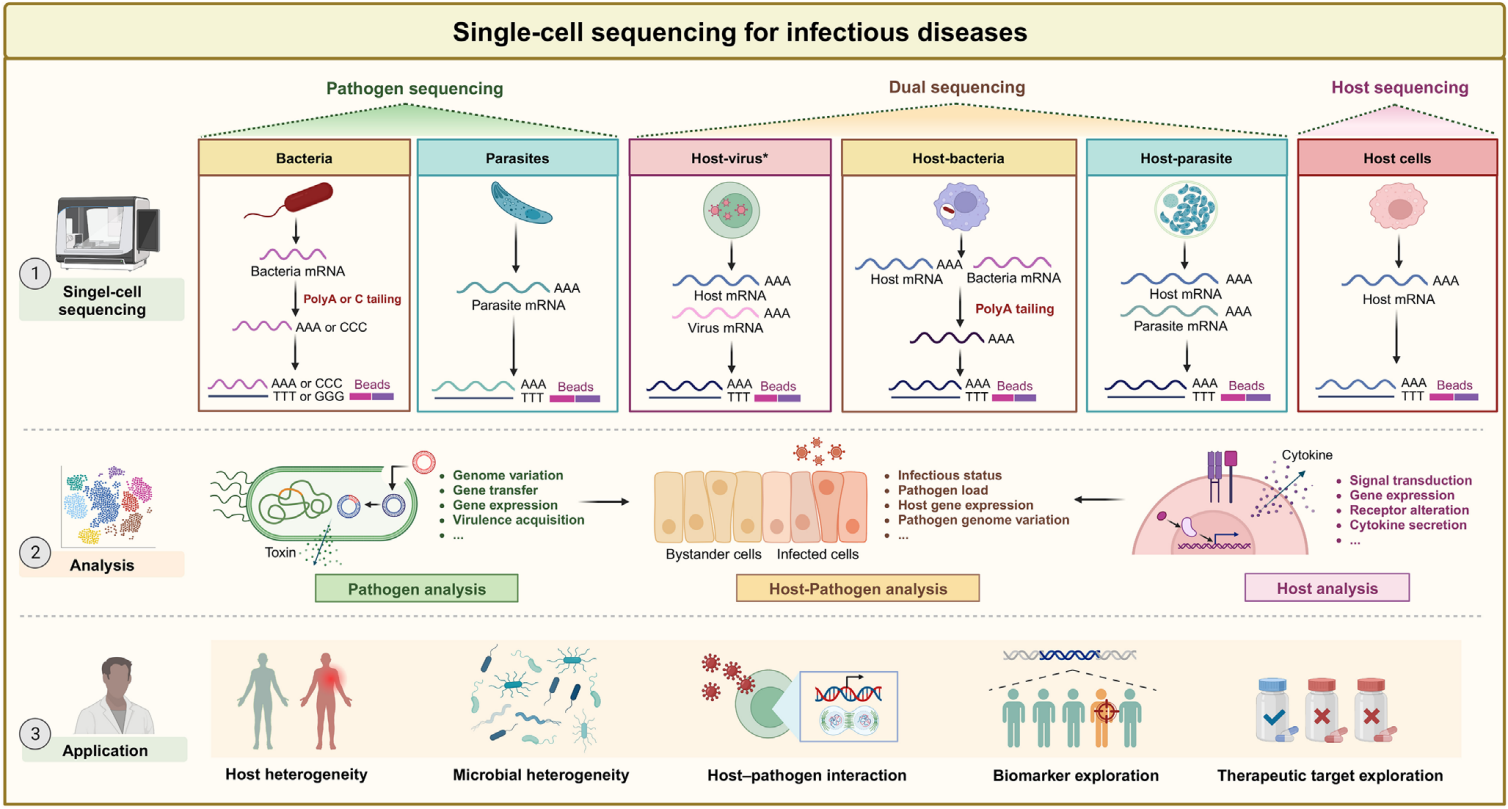
The development and application of single‐cell sequencing in infectious diseases. In infectious diseases, single‐cell sequencing technologies can be used to capture information about the pathogen, the host or both. The obtained data can be used for analyze the heterogenicity of pathogens (e.g., genomic variation and gene expression), the host (e.g., gene expression and ligand‐receptor interaction) and host‐pathogen interaction (e.g., intracellular pathogen load), thus facilitating the development of promising biomarkers or therapeutic targets. (Created with BioRender.com).^*^ This sequencing technology is suitable for viruses that acquire polyadenylate tails inside host cells. Abbreviation: ploy A: polyadenylate.

Single‐cell sequencing has rapidly advanced, including the development of multiple platforms at multi‐omics levels (genome, transcriptome, protein, etc.). The developmental history of single‐cell sequencing has been recorded.^[^ ^10^ ^]^ In infectious diseases, in addition to the host being a target for detection, pathogens can also be targeted for detection. Moreover, dual single‐cell sequencing technologies that can simultaneously obtain signals from both sides have also been developed. These technologies allow us to analyze the rich biological signals of microbial communities or host‐pathogen interactions, and solve the problem of one‐sided interpretation of infections from the perspective of the host (**Figure** **1**).

Eukaryotic single‐cell sequencing: The workflow includes four main steps: single‐cell isolation, library preparation, sequencing, and data processing.

To begin with, cell isolation is carried out using fluorescence‐activated cell sorting (FACS), microfluidic isolation, or laser capture microdissection (LCM). FACS, a high‐throughput screening method (>1000 cells/run), identifies specific surface markers to classify different cell populations by labeling cells with fluorescent monoclonal antibodies. This method is primarily suitable for blood and cell suspension. However, it may cause significant cell stimulation and require a large sample size. Microfluidic isolation is a microreactive system that includes three forms: including trap‐based microfluidics, valve‐based microfluidics, and droplet‐based microfluidics. During the process, a single cell is encapsulated in the inert carrier oil microdroplet, thus forming a closed space. This method allows high‐throughput screening (>1000 cells/run) of small sample sizes, while it requires sophisticated equipment and may also induce stimulation in certain cells.^[^ ^11^ ^]^ LCM uses a low‐energy infrared laser pulse to separate cells under a microscope, which can preserve both the morphological structure and spatial position information of cells, and therefore it is highly applicable to tissue samples.^[^ ^12^ ^]^ However, this method has a relatively low throughput and is prone to causing RNA contamination and gene loss. After isolation, physical (e.g., mechanical, thermal, and electrical methods) or chemical (e.g., enzymatic methods) methods are used for cell lysis.

Single‐cell library construction can randomly fragment long gene sequences into small pieces to cover the entire genome, and the chain‐specific library constructed by the transcriptome can retain mRNA orientation information, making gene expression quantification more accurate. Library construction can be divided into three parts: 1) Fragment DNA, end repair, and dA‐tailing; 2) ligation adaptor; and 3) sample index PCR. The principle of single‐cell library preparation is to obtain target materials and then construct library by amplification techniques (mainly whole‐genome amplification (WGA) and whole‐transcriptome amplification (WTA)). This step influences the sequencing accuracy and sensitivity largely and different strategies have been proposed. WGA refers to a biochemical strategy for achieving unbiased exponential amplification of the entire genomic content within a sample, which can be achieved using multiple annealing and looping‐based amplification cycles (MALBAC), degenerate oligonucleotide‐primed polymerase chain reaction (DOP‐PCR) and multiple displacement amplification (MDA). Details of these methods have been well described previously.^[^ ^13^ ^]^ And some new methods, such as rolling circle amplification (RCA), enable the analysis of extrachromosomal circular DNA and mitochondrial DNA at the single‐cell level.^[^ ^14^, ^15^ ^]^ WTA refers to a technology that amplifies RNA from a sample into sufficient quantities of complementary DNA (cDNA), primarily accomplished through two sequential steps: reverse transcription (two main ways: poly (A) tailing and template‐switching) followed by cDNA amplification (two main ways: PCR and in vitro transcription (IVT)). Different strategies are used in various methods. “Tang method”,^[^ ^16^ ^]^ an improved “Tang method”^[^ ^17^ ^]^ and CEL‐seq/CEL‐seq2^[^ ^18^, ^19^ ^]^ use ploy(A) tailing for reverse transcription, while Smart seq/Smart‐seq2 and single‐cell tagged reverse transcription (STRT)‐seq apply the template‐switching method. Except for CEL‐seq/CEL‐seq2, the techniques all utilize PCR to amplify cDNA, which is a nonlinear amplification process. And the amplification efficiency of PCR is sequence‐dependent. CEL‐seq/CEL‐seq2 uses IVT for amplification, which is a linear amplification process. IVT is performed after the second strand synthesis by adding a T7 promoter to the poly‐dT primers, thereby avoiding exponential amplification that may bias the gene expression profile towards shorter and less GC‐rich amplicons. To ensure the amplification efficiency and reduce the amplification bias, different techniques are also applied slightly differently in detail. For instance, Quartz‐seq, a further developed method from the improved “Tang method”, uses exonuclease I and suppression PCR to reduce unnecessary amplification by removing excess primers prior to second‐strand synthesis and generating hairpin structures, respectively.^[^ ^17^ ^]^ Smart seq/Smart‐seq2 uses M‐MLV reverse transcriptase and a template‐switching step to increase the abundance of full‐length transcripts. STRT‐seq applies the template‐switching method to add molecular barcodes and upstream primer‐binding sequences to achieve multiplex sequencing.^[^ ^20^ ^]^ More details of single‐cell RNA sequencing (scRNA‐seq) library preparation have been summarized.^[^ ^21^ ^]^

Then, sequencing can be performed, and the Illumina sequencing system is most commonly used in this step. Data processing generally includes primary analysis (base calling, demultiplexing, and alignment) and downstream analysis (dimensionality reduction, clustering, cell annotation, etc.). Then, analysis of genome variation, gene expression, functional enrichment, ligand‐receptor interactions, and others can be accomplished.

Of note, different sequencing strategies lead to variations in captured cell numbers, sequencing depth, and throughput. Detailed comparative results have been documented.^[^ ^22^, ^23^ ^]^ For example, the droplet‐based 5’ or 3’‐end sequencing platform (e.g., 10x Genomics) can offer gene expression profiles of about 20,000 cells in a single assay. Due to its high throughput and relatively shallow sequencing depth, it is well‐suited for studying highly heterogeneous tissues and large‐scale sampling of numerous cells. The plate‐based platforms (e.g., Smart‐seq2) have high read depth per cell but low throughput. Therefore, Smart‐seq2 can detect genes with low expression and is suitable for comprehensively characterizing a small cell population. Besides, Smart‐seq2 also provides full‐length transcript information, allowing splicing analysis.

Prokaryotic single‐cell sequencing: Bacteria belong to prokaryotes, and their non‐polyadenylated and shorter half‐life mRNA, relatively thick bacterial cell walls, and low abundance of genetic materials pose additional challenges to achieving prokaryotic single‐cell sequencing.^[^ ^24^ ^]^ Nowadays, several methods have been proposed to achieve prokaryotic single‐cell sequencing, including poly(A)‐independent multiple annealing and dC‐tailing‐based quantitative scRNA‐seq (MATQ‐seq),^[^ ^25^, ^26^ ^]^ tagging RNA In Situ and sequencing (PETRI‐seq),^[^ ^27^ ^]^ microbial split‐pool ligation transcriptomics (microSPLiT),^[^ ^28^ ^]^ BacDrop,^[^ ^29^ ^]^ and so on.^[^ ^30^ ^]^ Based on these advances, details of bacterial behaviors, such as the growth‐dependent transcriptomes of individual *Salmonella* and *Pseudomonas*,^[^ ^25^ ^]^ relationships between cell states and growth phases of *Escherichia coli*,^[^ ^27^ ^]^ heterogeneous responses driven by mobile genetic elements (e.g., IS*903B*) in *Klebsiella pneumoniae* under antibiotic stress,^[^ ^29^ ^]^ are revealed, allowing us to understand these pathogens at a finer level.

The overall workflow of prokaryotic single‐cell sequencing is similar to that of eukaryotic sequencing, while there are certainly some differences. MATQ‐seq, PETRI‐seq, and microSPLiT are plate‐based methods, while BacDrop is a droplet‐based method. For plate‐based methods, taking MATQ‐seq as an example, FACS and enzymatic methods are used to separate individual bacteria onto a plate and lyse bacteria, respectively. To capture low‐copy RNA, random primer‐induced reverse transcription and multiple annealing are then performed. After poly(C) tailing and Tn5 transposase‐induced cDNA labeling, sequencing libraries are generated, and sequencing can be performed.

For BacDrop, its uniqueness mainly lies in reverse transcription and barcoding. After rRNA and gDNA removal in fixed and permeabilized cells, the addition of CB1 and UMIs to cDNA (first barcoding) and cDNA polyadenylation are performed in cells. Then, the second strand cDNA synthesis that is induced by the poly(A) tail of cDNA, and the addition of CB2 (second barcoding) and capturing cycles are performed. After cDNA purification and amplification, tagmentation and enrichment, libraries are constructed and sequencing is carried out.

Dual single‐cell sequencing: Due to the inherent characteristics of pathogens, dual single‐cell sequencing of viruses or parasites alongside the host is relatively easier to accomplish.^[^ ^31^, ^32^ ^]^ However, the differences in genetic material abundance and structures between bacteria and the host exacerbate the difficulties of dual single‐cell sequencing for bacterial infections. Currently, two teams are developing dual scRNA‐seq technologies for bacterial infections, allowing for the assessment of their respective states.^[^ ^33^, ^34^ ^]^ To be specific, Avital G et al.^[^ ^33^ ^]^ capture the transcriptomic information of *Salmonella*‐infected macrophages based on their newly developed method. In this method, FACS and enzymatic methods are used for cell sorting and lysis, respectively. During the RNA amplification stage, random hexamers are used for reverse transcription, followed by RNase treatment and poly(A) tailing. Next, CEL‐Seq2 barcoded primers are applied in the second strand synthesis, and then linear amplification by IVT is conducted after pooling the samples. In the library preparation stage, random primers are used for reverse transcription of amplified RNA, and PCR is then performed. Paired‐end Illumina sequencing is conducted, and sequencing data can be processed by the CEL‐Seq2 pipeline. Notably, the number of bacterial genes detected by this method is limited when bacterial RNA abundance is low.

To address the above question, Betin V et al.^[^ ^34^ ^]^ develop a technique, named pathogen hybrid capture (PatH‐Cap), to enrich bacterial mRNA and exclude bacterial rRNA and tRNA. The key to PatH‐Cap is to design the appropriate probes. The probes consist of 100bp sequences tiled along the target bacterial sequence (including coding mRNA and annotated non‐coding RNA, but not tRNA and rRNA). Single‐stranded, biotinylated probes are generated from the probe templates using IVT and incubated with sequencing libraries constructed based on the protocol of Avital G et al.^[^ ^33^ ^]^ Then, bacterial mRNA targets are pulled down with streptavidin‐coated beads, and Post‐PatH‐Cap libraries are generated through PCR amplification. Libraries before and after PatH‐Cap treatment undergo paired‐end sequencing to obtain host‐ and bacteria‐rich transcript information. This approach enables single‐cell analysis of low‐input samples (e.g., cells infected with 1–3 *Pseudomonas aeruginosa*).

The comparisons of some common scRNA‐seq platforms are summarized in **Table** **1**.

**Table 1:** The comparisons of some common single‐cell RNA sequencing platforms.

	Platforms	Isolation methods	Cell numbers^*)^	Sensitivity^*)^	Amplification methods	UMI^a)^	Region	Refs.
Eukaryotic single‐cell RNA sequencing	Smart‐seq2^b)^	FACS^c)^	10^2	≈9,138 genes/cell (median)	PCR^d)^	No	Full‐length	[35, 36]
	10x Genomics	Microfluidic	10^3–10^4	500‐1,500 genes/cell	PCR	Yes	3’ end	[37]
	SPLiT‐seq^e)^	Split‐pool barcoding^**)^	≈10^5	1,000 ‐10,000 RNAs/cell	PCR	Yes	3’ end	[38]
	mDrop‐seq^f)^	Microfluidics	≈10^4	200‐2,000 genes/cell	PCR	Yes	3’ end	[39]
	Drop‐seq	Microfluidics	≈10^4	≈4,811 genes/cell (median)	PCR	Yes	3’ end	[36, 40]
	inDrops^g)^	Microfluidics	≈10^4	≈1,000,000 reads/cell	IVT^h)^	Yes	3’ end	[41]
	SCRB‐seq^i)^	FACS	>10^3	≈7,906 genes/cell (median)	PCR	Yes	3’ end	[36, 42, 43]
	ICO‐seq^j)^	Microfluidic	>10^2	≈15,000‐20,000 mRNAs/cell	PCR	Yes	3’ end	[44]
	CEL‐Seq^k)^	FACS	>10^2	≈1,000 genes/cell	IVT	Yes	3’ end	[19]
	CEL‐Seq2	FACS	>10^2	≈7,536 genes/cell (median)	IVT	Yes	3’ end	[18, 36]
	MARS‐Seq^l)^	FACS	> 10^3	≈4,763 genes/cell (median)	IVT	Yes	3’ end	[36, 45]
	Quartz‐Seq2	FACS	≈10^4	0.19 M fastq reads/10 pgRNA	PCR	Yes	3’ end	[46]
	BD Rhapsody	Microwells	> 10^4	≈45,000 reads/cell	PCR	Yes	3’ end	[47]
	YscRNA‐seq^m)^	FACS	≈10^2	≈3,400 genes/cell	PCR	Yes	5’ end	[48]
	Cyto‐Seq^n)^	Microfluidic	≈10^5	20,000 to 500,000 reads /cell	PCR	Yes	3’ end	[49]
Prokaryotic single‐cell RNA sequencing	PETRI‐seq^o)^	Split‐pool barcoding^**)^	≈10^4	>200 mRNAs/cell	PCR	Yes	3’ end	[27]
	MicroSPLiT^p)^	Split‐pool barcoding^**)^	>10^4	100‐300 mRNA transcripts /cell	PCR	Yes	3’ end	[28]
	BacDrop^q)^	Microfluidic	>10^4	≈80,000 reads/cell	PCR	Yes	3’ end	[29]
Dual single‐cell RNA sequencing	CEL‐Seq2	FACS	>10^2	≈7,536 genes/cell (median)	IVT	Yes	3’ end	[18, 36]
	MATQ‐seq^r)^	FACS	≈100	≈18,354 genes/eukaryotic cell ≈300‐600 genes/prokaryotic cell	PCR	Yes	Full‐length	[50, 51, 52]
	scDual‐Seq^s)^	FACS	≈100	≈470 mRNAs/cell	PCR	Yes	3’ end	[33]
	PatH‐Cap^t)^	FACS	≈100	≈175 genes/cell	IVT	Yes	3' end	[34]
	Seq‐Well	Microfluidics	10^3–10^4	≈6,927genes/ cell	PCR	Yes	3’ end	[53, 54]

*) Due to different experimental conditions, the number of captured cells and the detection sensitivity may fluctuate.

**) Using barcoding as an alternative to cell sorting.

^a)^ unique molecular identifier;

^b)^ switching mechanism at 5’end of the RNA transcript sequencing;

^c)^ fluorescence‐activated cell sorting;

^d)^ polymerase chain reaction;

^e)^ split pool ligation‐based transcriptome sequencing;

^f)^ microbial drop‐seq;

^g)^ indexing droplets;

^h)^ in vitro transcription;

^i)^ single‐cell RNA barcoding and sequencing;

^j)^ isogenic colony sequencing;

^k)^ cell expression by linear amplification and sequencing;

^l)^ massively parallel single‐cell RNA‐seq;

^m)^ yeast single‐cell RNA‐seq;

^n)^ cytometry single‐cell RNA‐seq;

^o)^ prokaryotic expression profiling by tagging RNA in situ and sequencing;

^p)^ microbial split‐pool ligation transcriptomics sequencing;

^q)^ bacterial droplet‐based single‐cell RNA‐seq;

^r)^ multiple‐annealing‐and‐tailing‐based quantitative scRNA‐seq in droplets;

^s)^ single‐cell dual RNA sequencing;

^t)^ pathogen hybrid capture.

## 3 Applications of Single‐Cell Sequencing in Infectious Diseases

The interactions between pathogens and hosts are highly complex, involving processes such as pathogen invasion, host defense and pathogen immune evasion (Figure S1, Supporting Information). Single‐cell sequencing has further deepened our understanding of the above process. In the following part, we summarize the multifaceted applications of this technology in infectious diseases. Relevant evidence is presented according to the type of infection (prion, mycoplasma, viral, bacterial, fungal and parasitic infections). For each type of infection, we select some common, high‐burden or high‐concern diseases to provide detailed summaries and discussions. As for other infectious diseases, we present them in a table format. We also listed Table S1 (Supporting Information) to summarize some potential therapeutic targets for infectious diseases discovered based on single‐cell sequencing.

### 3.1 Prion Infections/Prion Diseases

Prion diseases are lethal neurodegenerative disorders affecting both humans and animals, and their pathogenic factor is scrapie prion protein (PrP^Sc^). PrP^Sc^ that can be transmitted between individuals is a misfolded conformation of cellular prion proteins (PrP^C^),^[^ ^55^ ^]^ and its accumulation in the central nervous system is accompanied by neuronal loss, spongiform changes, and gliosis, which are considered related to the pathogenesis of prion diseases.^[^ ^56^ ^]^ Although several potential mechanisms (the toxicity of PrP^Sc^, the loss of brain cells, etc.) have been proposed, the pathogenesis of prion diseases is currently subject to debate.^[^ ^57^ ^]^ Potential heterogeneity among different brain cells has been reported during prion disease,^[^ ^58^, ^59^ ^]^ emphasizing the necessity of utilizing single‐cell sequencing technologies to analyze the behavior of distinct cell populations and identify disease‐crucial molecules.

Slota JA et al.^[^ ^57^ ^]^ provide a single‐cell landscape of prion diseases based on prion‐infected mice. In detail, genes related to brain homeostasis (lipid synthesis, glutamate clearance, etc.) are transcriptionally inactive in astrocytes. Among these astrocytes, disease‐depleted subpopulations are considered to be neuroprotective astrocytes characterized by altered expression of homeostasis‐related genes. While an increased subpopulation‐8 may be reactive astrocytes with low expression of homeostatic/neuroprotective genes, signifying a loss of their neuroprotection. Moreover, the diversity of microglial activation states (proliferating, phagocytic, interferon (IFN) responding, etc.) is described. This single‐cell landscape exhibits the complexity of prion diseases and provides a basis for identifying pathological cells or molecules.

### 3.2 Mycoplasma Infections

*Mycoplasma pneumoniae*, the agent of Mycoplasma pneumoniae pneumonia, is a major human pathogen primarily responsible for lower respiratory tract infections of varying severity. To investigate the underlying mechanisms behind the varying disease severity among patients, Shen X et al.^[^ ^60^ ^]^use scRNA‐seq to profile the immune landscape of bronchoalveolar lavage fluid from children with Mycoplasma pneumoniae pneumonia of different severity. They find that the levels of exhausted T cells and M1‐like macrophages are positively related to severity. And the exhaustion status of T cells can be induced by persistent IFN‐I signaling and inadequate assistance of CD4+ T cells. Also, the enhanced cell–cell interactions between exhausted T cells and programmed death‐ligand 1+ macrophages are found in severe patients, which may be responsible for the dysfunction of anti‐*Mycoplasma pneumoniae* immune responses. Together, this work deepens our understanding of Mycoplasma pneumoniae pneumonia and provides some potential therapeutic targets.

### 3.3 Viral Infections

In this section, COVID‐19‐related evidence has been well summarized,^[^ ^61^ ^]^ and therefore, our main focus is on HIV infection/AIDS, viral hepatitis, herpes, and influenza. Evidence related to other viral infections is summarized in **Table** **2**.

**Table 2:** Evidence related to other viral infections.

Infection or disease	Focus	Refs.
BKPyV^a)^ infections	The characteristics of broadly neutralizing antibodies in BKPyV infection	[62, 63]
	The transcriptomic atlas of primary human renal proximal tubular epithelial cells in BKPyV infection	[64]
Cytomegalovirus infections	The characteristics of natural killer cells in cytomegalovirus infection	[65, 66]
Dengue virus infections	The transcriptomic and clonality atlas of dengue virus infection	[67, 68, 69, 70]
	The characteristics of broadly neutralizing antibodies in dengue virus infection	[71]
Ebola virus infections	The immune atlas of Ebola virus infection	[5]
	The long non‐coding RNA atlas of Ebola virus infection	[72]
	The composition of the Glycoprotein‐reactive antibody repertoire in Ebola virus infection	[73]
Foot‐and‐mouth disease	The characteristics of neutralizing antibodies in enterovirus A71 infection	[74]
	The mechanisms of persistent foot‐and‐mouth disease virus infection	[75]
Hantaan virus infections	The role of erythrocytes in Hantaan virus infection	[76]
HCV^b)^ infections	The characteristics of HCV‐specific T cells	[77, 78, 79]
	The T cell receptor repertoire atlas in different infection stages	[80]
	The myeloid cell atlas before and after anti‐HCV treatment	[81]
	The transcriptomic atlas of T cells before, during and after antiviral therapy	[82]
	The transcriptomic and B cell receptor repertoire atlas during chronic infection	[83]
HASTV^c)^ infections	The transcriptomic atlas of HASTV‐induced intestinal infection	[84]
HPV^d)^ infections	The relationship between HPV life cycle and epidermal hyperplasia	[85]
HSV‐2^e)^ infections	The transcriptomic atlas of HSV‐2 infection.	[86, 87]
HTLV‐1^f)^ infections	The multi‐omics atlas of premalignant cells and the multicellular ecosystem	[88]
	The transcriptomic or cell receptor atlas of HTLV‐1 infection	[89, 90]
	The mechanism of HTLV‐1‐mediated transformation and immune escape	[91]
IVB^g)^ infections	The transcriptomic atlas of IVB infection	[92]
JHMV^h)^ infections	The transcriptomic atlas of JHMV‐induced central nervous system infection	[93]
	The role of microglia in neurotropic coronavirus infection	[94]
KSHV^i)^ infections	The transcriptomic atlas of KSHV infection	[95, 96, 97, 98]
	The mechanism of caspase inhibition mediated antiviral status	[99]
LCMV^j)^ infections	The transcriptomic or clonal atlas of CD4+ T cells in different infection stages	[100]
	The transcriptomic atlas of CD4+ T cells in self‐resolving and chronic infections	[101, 102, 103]
	The differentiation mechanism of memory and follicular helper CD4+ T cells	[104]
	The atlas of exhausted CD8+ T cells in chronic infection	[105]
	The transcriptomic and antibody repertoire atlas of plasma cells in chronic infection	[106]
	The role of myeloid cells in shaping viral response	[107]
	The role of Trib1^k)^ in chronic infection	[108]
Measles virus infections	The mechanism of Measles virus replication in airway epithelia	[109]
Norovirus infections	The response of human intestinal cell lines to infection and the mechanism of viral replication	[110]
NTHi^l)^ infections	The role of heparin‐binding epidermal growth factor in the hyperplasia of middle ear	[111]
Rabies	The transcriptomic and spatial atlas during infections	[112, 113, 114, 115]
Reovirus infections	The transcription and spatial atlas of reovirus‐induced myocarditis	[116]
	The regenerative transcriptional response of the intestinal epithelium in rotavirus infection	[117]
Respiratory syncytial virus infections	The transcriptomic atlas of primary bronchial epithelial cells during infection	[118]
	The immune responses triggered by the chimeric respiratory syncytial virus vaccine	[119]
Rhinovirus infections	The rhinovirus‐specific B cell responses	[120]
RVFV^m)^ infections	The brain immune response in RVFV infection	[121]
	The characteristics of neutralizing antibody	[122]
SARS^n)^	The transcriptomic atlas of SARS	[123]
Sendai virus infections	The role of alveolar type 1 cells in the repair of Sendai virus‐infected lung injury	[124]
	Ligand‐receptor interactions in lungs	[125]
	The role of basal epithelial cells in Sendai virus‐induced lung disease	[126]
SFTSV^o)^ infections	The transcriptomic atlas of SFTSV infection	[127, 128]
	The relationship between NETosis and SFTSV infection	[129]
VEEV^p)^ infections	The transcriptomic atlas of VEEV infection	[130, 131]
Yellow fever virus infections	The clonal phenotypes of T cells in acute viral infections	[132]
VSV^q)^ infections	The genetic structure and diversity of VSV	[133]
West Nile virus infections	The transcriptomic atlas of microglia and monocyte‐derived cells	[134]
	The transcriptomic atlas of antiviral response	[135]
	The relationship between microglia and CD8+ T cells	[136]
	The evolution of neutralizing antibodies from natural infection	[137]
Zika virus infections	The immune atlas of Zika virus infection	[138]
	The temporal and cellular atlas of Zika virus fetal brain infection	[139]
	The transcriptomic atlas of Zika virus‐induced testicular injury	[140, 141]
	The roles of fetal monocytes/macrophages and microglia in Zika dissemination and clearance	[142]
	The immune response induced by different Zika virus strains	[143]
	The immune impact of prior dengue virus infection on secondary Zika infection	[69, 144]

^a)^ BK polyomavirus;

^b)^ hepatitis C virus;

^c)^ human astrovirus;

^d)^ human papilloma virus;

^e)^ herpes simplex virus type 2;

^f)^ human T cell leukemia virus type 1;

^g)^ Influenza B virus;

^h)^ JHM strain of mouse hepatitis virus;

^i)^ Kaposi's sarcoma‐associated herpesvirus;

^j)^ lymphocytic choriomeningitis virus;

^k)^ Tribbles pseudokinase 1;

^l)^ nontypeable haemophilus influenzae;

^m)^ Rift Valley fever virus;

^n)^ severe acute respiratory syndrome;

^o)^ severe fever with thrombocytopenia syndrome virus;

^p)^ Venezuelan equine encephalitis virus;

^q)^ vesicular stomatitis virus.

#### 3.3.1 HIV Infection/AIDS

**Figure 2:**
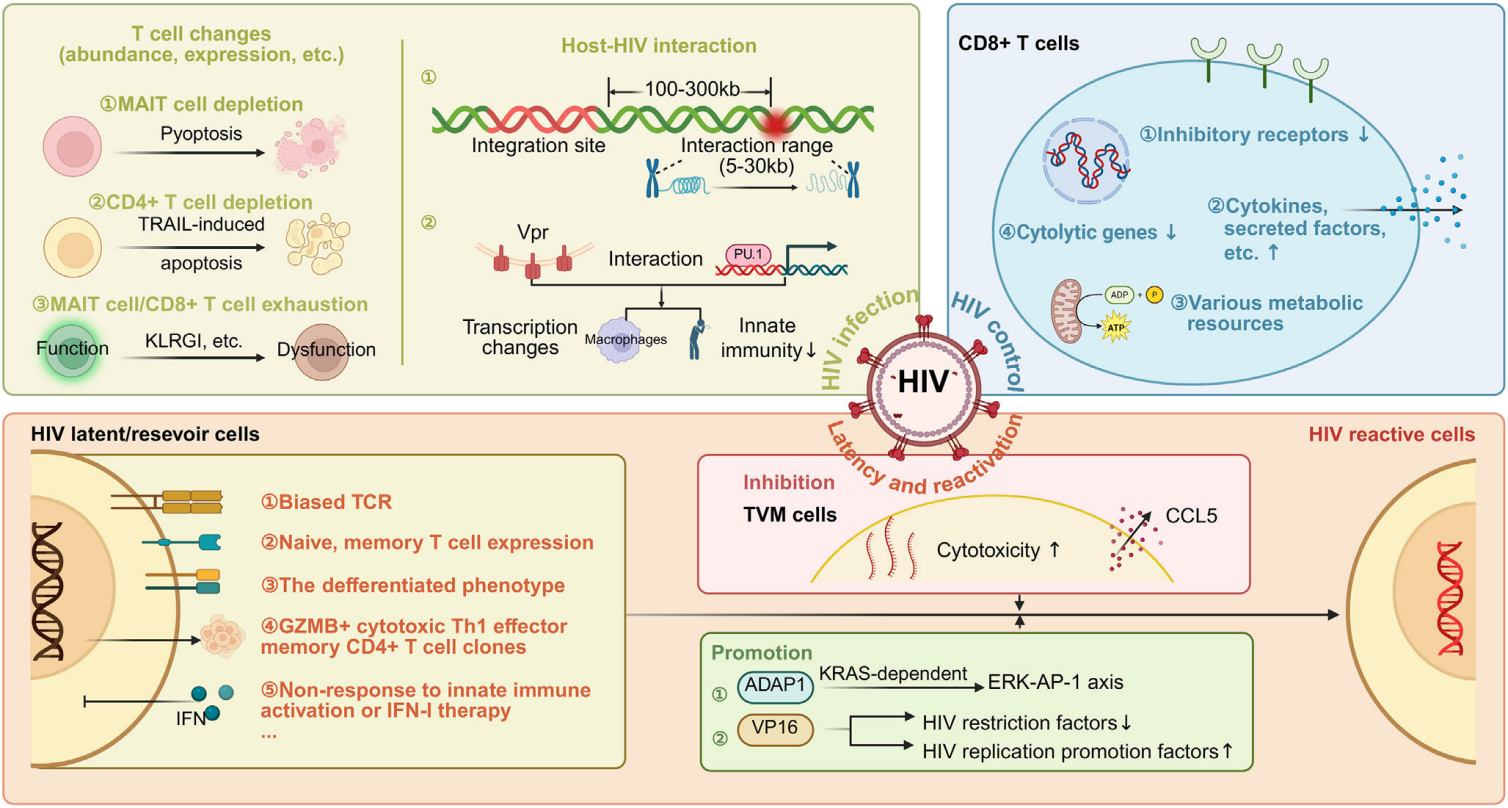
The characteristics of HIV infection/AIDS at the single‐cell level. During HIV infection/AIDS, the abundance, gene expression, and cell receptors of T cells are changed. The host can interact with HIV in various ways to regulate gene expression, innate immunity, and other processes. HIV latent cells show many specific characteristics, including biased TCR, a differentiated phenotype, and others. There are many mechanisms that mediate the transition from HIV latency to reactivation (e.g., ADAP1‐induced ERK‐AP‐1 axis). However, TVM cells inhibit the above transformation in a CCL5‐dependent manner. The host can control HIV infection through a variety of ways, and CD8+ T cells are the main force in controlling HIV. They achieve anti‐HIV function through downregulating inhibitory receptors and cytolytic genes, increasing cytokines, and other approaches. (Created with BioRender.com).

HIV, a retrovirus, is the causative agent of AIDS and can be transmitted through body fluids and secretions. HIV targets host cells, especially CD4+ T cells, to destroy them and thus induces host immunosuppression. Such immunosuppression can cause the host to suffer from opportunistic infections, autoimmune diseases, and malignancies. An estimated 38 million individuals worldwide are living with HIV, and such a heavy burden may be partly attributed to the incurable nature of this infection.^[^ ^145^ ^]^ Decoding the incurable nature of HIV infection requires understanding how HIV establishes infection and responds to treatment, how it maintains latency, how reactivation occurs, and the approaches by which host cells control or eliminate HIV. HIV is an intracellular pathogen, and therefore, exploring cellular immunity (cellular dynamics, cellular communications, etc.) is crucial to answering these previous questions. The single‐cell sequencing technology has emerged as an important tool in the field of HIV infection/AIDS due to its unique advantages in analyzing multicellular processes. HIV infection/AIDS‐related evidence is shown in **Figure** **2**.

1) The immune profiles and pathogenesis of HIV infection/AIDS

In terms of HIV infection/AIDS‐relevant events, host changes in different tissues are captured.^[^ ^4^, ^146^, ^147^, ^148^, ^149^, ^150^, ^151^, ^152^, ^153^, ^154^, ^155^, ^156^, ^157^, ^158^, ^159^, ^160^, ^161^, ^162^, ^163^, ^164^, ^165^, ^166^, ^167^, ^168^, ^169^, ^170^, ^171^, ^172^, ^173^, ^174^, ^175^, ^176^, ^177^, ^178^, ^179^, ^180^, ^181^ ^]^ For instance, during infection, the abundance of CD8+ effector memory T cells is increased,^[^ ^147^ ^]^ while the amount of CD101‐expressing CD4+ T cells is reduced.^[^ ^158^ ^]^ Indeed, T cell abundance changes are a typical feature of HIV infection. Single‐cell technologies allow us to delve deeper into the mechanisms behind these typical changes. Xia P et al.^[^ ^182^ ^]^ note that mucosal‐associated invariant T (MAIT) cells show a pyroptotic phenotype with upregulation of pyroptosis‐related genes during infection, and propose that enhanced pyroptosis in MAIT cells is responsible for their loss. Other reports find that the augmented expression of TRAIL during infection can modulate the apoptosis of CD4+ T cells and, thus their depletion,^[^ ^149^, ^183^, ^184^ ^]^ and therefore, the TRAIL may serve as a target to restore HIV‐induced CD4+ T cell loss.^[^ ^149^ ^]^ Additionally, both infected cells and uninfected bystander cells can express the short TRAIL isoform that can act as an agonist for TRAIL‐mediated killing.^[^ ^185^ ^]^ And some agents (IFN‐I, Toll‐like receptor (TLR) 7 agonist, etc.) can stimulate the generation of this short isoform, indicative of a novel strategy against TRAIL‐mediated biological processes.^[^ ^185^ ^]^

There are also dramatic changes in the transcriptome during infection (e.g., increased LAT2 expression).^[^ ^186^ ^]^ The team of Collora JA applies single‐cell omics technologies to simultaneously capture HIV‐ and host‐derived signals and thus explains the regulatory mechanism behind HIV‐related transcriptional changes. HIV can interact with the host at a distance of about 100–300 kb from integration sites, leading to enhanced host chromatin accessibility within the range of 5–30 kb and thus influencing the transcriptional pattern.^[^ ^187^ ^]^ ETS, RUNT, and ZNF‐family transcription factors can act as mediators for such interactions.^[^ ^188^ ^]^

The loss of effector function is also one of the mechanistic of HIV infection. KLRG1 and IFN‐α or IL‐10 receptors are identified as the promoting factors of CD8+ T cell exhaustion and MAIT cell dysfunction, respectively, which may serve as reliable exhaustion recovery targets.^[^ ^189^, ^190^ ^]^ Certainly, there are other mechanisms by which HIV benefits itself, including reducing the expression of host innate immunity‐related transcription factors.^[^ ^191^, ^192^ ^]^

2) The characteristics of latent or reservoir cells

HIV persistence and latency are primarily responsible for its incurable characteristics.^[^ ^145^, ^185^ ^]^ Reservoirs mainly contribute to HIV persistence, which refers to cells or anatomical sites that contain replicating forms of HIV.^[^ ^193^ ^]^ An important source of the reservoir is latently infected cells. These latently infected cells refer to a reversible, non‐productive state of infection in individual cells. Although these cells do not generate infectious virus particles, they retain the ability to do so.^[^ ^193^ ^]^ Nowadays, four potential mechanisms that facilitate the establishment or maintenance of HIV latency have been reported, including the epigenetic regulation of HIV transcription, the location of the integration site, the influence of microRNAs and the accessibility of transcription factors.^[^ ^194^ ^]^ However, latently infected cells or reservoir cells exhibit great heterogeneity in cell types, cell status, transcriptomic profiles and others,^[^ ^6^ ^]^ which may hinder the effectiveness of therapeutic strategies developed based on the aforementioned mechanisms. In such circumstances, the necessity of application of single‐cell sequencing technologies is emphasized again. Latent or reservoir CD4+ T cells have been typified by a biased T‐cell receptor (TCR) repertoire,^[^ ^8^ ^]^ a dedifferentiated phenotype,^[^ ^195^ ^]^ non‐response to innate immune activation or IFN‐I therapy,^[^ ^196^ ^]^ and so on.^[^ ^9^, ^197^, ^198^, ^199^, ^200^, ^201^, ^202^, ^203^, ^204^, ^205^ ^]^ These signature molecules not only provide a breakthrough in understanding the mechanisms of HIV latency and persistence, but also serve as targets for killing HIV. This may potentially change the situation where HIV infection/AIDS is considered incurable.

3) The mechanism of HIV reactivation

Under optimal conditions (a larger cell size, G1 phase of host cells, high levels of transcriptional activators, etc.),^[^ ^206^, ^207^ ^]^ HIV reactivation from latency occurs, and there is also heterogeneity in this state transition process. Cortés‐Llanos B et al.^[^ ^186^ ^]^ reveal two different reactivation phenotypes of T cells (the fast and slow reactivation phenotypes) and further discover the transcriptional differences of these T cells with diverse phenotypes, especially in the expression of genes related to inflammation, immune activation, and host cellular and viral transcription factors (e.g., TCF7 and HTATSF1). Hu W et al.^[^ ^208^ ^]^ also, focus on this topic and report the differences in the ability of memory T cell subsets to regulate HIV reactivation based on scRNA‐seq data. They further divide memory T cells as CCL4+CCL5‐ and CCL4‐CCL5+ central memory cells and find that virtual memory CD8+ T (TVM) cells are enriched in CCL4‐CCL5+ central memory cells. These TVM cells have enhanced T‐bet and RUNX3‐driven cytotoxicity and can powerfully suppress HIV reactivation in a CCL5‐dependent manner compared to non‐TVM cells.^[^ ^208^ ^]^ Another study points out that these TVM cells have a specific metabolic feature with active glutamate metabolism and both glycolysis‐ and oxidative phosphorylation‐dependent energy supply.^[^ ^209^ ^]^ In vitro, upregulated glutamate has been shown to damage the anti‐HIV ability of TVM cells through the mechanistic target of rapamycin complex 1 pathway, indicative of the potential of glutamate as a target for regulating TVM cell function. Additionally, the roles of FOXP1 and GATA3 in HIV reactivation are also reported.^[^ ^210^ ^]^

Additionally, scRNA‐seq also reveals the roles of ADAP1 and VP16 in HIV reactivation. ADAP1 can manipulate the transcriptome of T cells, particularly genes containing cis‐elements enriched for canonical TCR‐induced transcription factors, to facilitate HIV reactivation through activation KRAS‐dependent ERK‐AP‐1 axis.^[^ ^211^ ^]^ VP16, a herpes simplex virus type 2 (HSV‐2) protein that modulates transcription, is able to promote the expression of MALAT1 that can drive HIV replication and inhibit the restriction factor of HIV transcription (H3K27me3), thus contributing to HIV reactivation.^[^ ^212^ ^]^ The aforementioned studies provide crucial evidence for the development of new strategies to inhibit HIV reactivation.

4) Host response to HIV infection/AIDS

To respond HIV invasion, host cells mount a powerful counterattack, which can manifest as either HIV control or elimination. CD8+ T cells contribute greatly to long‐lasting HIV control, and these cells can be featured by downregulation of inhibitory receptors and cytolytic genes (e.g., TIGIT),^[^ ^213^ ^]^ increased cytokines,^[^ ^213^ ^]^ and various metabolic resources.^[^ ^214^ ^]^ Apart from CD8+ T cells, CD64^Hi^ PD‐L1^Hi^ myeloid DCs, CD14+ DCs, and IL‐27‐treated monocyte‐derived macrophages with anti‐HIV capacity are discovered by scRNA‐seq.^[^ ^215^, ^216^, ^217^ ^]^ Molecularly, the beneficial roles of IL‐15 and Prothymosin α in HIV control or elimination are also reported.^[^ ^7^, ^214^ ^]^

Abbreviation: MAIT: mucosal‐associated invariant T; HIV: human immunodeficiency virus; TCR: T cell receptor; Th1: T helper type 1; IFN‐I: type I interferon; TVM cells: virtual memory CD8+ T cells

#### 3.3.2 Hepatitis B

Hepatitis B virus (HBV) is the pathogen of hepatitis B, causing a global health catastrophe. Over one‐third of the population is estimated to be exposed to HBV, resulting in 257 million chronic infections.^[^ ^218^ ^]^ Hepatitis B is considered a highly heterogeneous disease that is featured by variable viral burden and liver inflammation.^[^ ^219^ ^]^ The infection progresses through four clinical and/or virological stages, culminating in a functional cure stage where the levels of quantitative (q)HBsAg and covalently closed circular DNA (cccDNA) decrease over time. However, HBV is still present, and the persistence of cccDNA in the liver can mediate spontaneous or iatrogenic viral reactivation.^[^ ^220^ ^]^ Analyzing the behaviors of different cells is the basis for understanding the mechanism of disease stage transition.

1) The immune profiles of hepatitis B patients with different characteristics

Based on single‐cell sequencing data, the diverse responses of patients with different characteristics are explored.^[^ ^219^, ^221^, ^222^, ^223^, ^224^, ^225^, ^226^, ^227^, ^228^, ^229^, ^230^, ^231^, ^232^ ^]^ For instance, in immune active patients, exhausted CD8+ T cells are derived from liver‐resident GZMK+ effector memory T cells and self‐expansion, while in acute recovery patients, they originate from blood CX3CR1+ effector T cells and GZMK+ effector memory T cells.^[^ ^223^ ^]^ Other perturbations of cell populations under drug stress are also depicted.^[^ ^233^, ^234^, ^235^, ^236^, ^237^ ^]^

2) The pathogenesis of hepatitis B

B cell depletion can impair T cell function and lead to delayed HBV clearance. The effect of B cells on T cells may be mediated by B cell antigen presentation and co‐stimulatory functions. IFN‐α plays a positive regulatory role in the interaction between B cells and T cells and may be a potential target for anti‐HBV treatment.^[^ ^237^ ^]^

Moreover, neutrophil extracellular traps (NETs), the web‐like structures released by activated neutrophils, also function in HBV infection. Increased NETs are related to coagulation dysfunction in HBV patients with acute liver injury. Mechanically, NETs exacerbate liver injury in fulminant viral hepatitis by facilitating fibrin deposition and amplifying the inflammatory response. And NET is formed through the interaction of fibrinogen‐like protein 2 (FGL2) and mucolipin 3 to regulate calcium influx and activate autophagy. NET may serve as a potential therapeutic target for treating fulminant viral hepatitis‐related liver injury.^[^ ^238^ ^]^ HBV infection can also enhance viral infectivity by upregulating host neuropilin‐1 expression and facilitating the interaction between neuropilin‐1 and the preS1 domain of large hepatitis B surface proteins.^[^ ^239^ ^]^

#### 3.3.3 Herpesvirus Infections

Herpesviruses are a group of enveloped DNA viruses that are primarily categorized into three types: α‐herpesviruses, β‐herpesviruses, and γ‐herpesviruses. α‐herpesviruses encompass herpes simplex virus types 1 (HSV‐1) and HSV‐2, along with the varicella‐zoster virus (VZV). The predominant β‐herpesvirus is human cytomegalovirus (HCMV), while γ‐herpesviruses primarily consist of the Epstein‐Barr virus (EBV) and Kaposi's sarcoma‐associated herpesvirus (KSHV).^[^ ^240^ ^]^ In each type (α‐, β‐, and γ‐herpesviruses), the representative viruses (such as HSV‐1) are selected for detailed discussion. Literature related to infections caused by other herpesviruses is listed in Table 2.

α‐herpesviruses infections:

HSV‐1 infections: HSV‐1 is a widely distributed neurotropic double‐stranded DNA virus, and it is transmitted through close contact between infected and susceptible individuals, causing labial, ocular, or genital infections. After primary infection, HSV‐1 establishes latency in neurons of the peripheral nervous system. It can periodically reactivate throughout the host's lifetime, triggering repeated clinical or subclinical episodes.^[^ ^241^ ^]^ Why do individuals and cells exhibit differences in susceptibility to HSV‐1? Why does HSV‐1 activate repeatedly to cause recurrent episodes in some hosts but not in others? The molecular mechanisms underlying such heterogeneity require single‐cell sequencing technologies to provide answers.

1) The pathogenesis of HSV‐1 infections

Based on scRNA‐seq, Wang Y et al.^[^ ^242^ ^]^ find that YY1‐induced MAMDC2‐AS1 upregulation can promote the nuclear transport of viral tegument protein VP16 and the expression of HSV‐1 immediate‐early genes through interaction with Hsp90α, thus increasing host susceptibility. Additionally, HSV‐1 can recruit β‐catenin to the host cell nucleus and viral replication sites, facilitating late viral gene expression and progeny production.^[^ ^243^ ^]^ HSV‐1 also simulates an embryonic‐like transcriptional program to prevent epigenetic silencing of its genome and promote its gene expression.^[^ ^244^ ^]^ The substantial pathway by which HSV‐1 mediates the inflammatory process by activating TNFSF members even under the pressure of antiviral drugs is also reported.^[^ ^245^ ^]^

2) The host response to HSV‐1 infections

While the host can take a series of measures to counter HSV‐1 attacks. NRF2, Gadd45b, IRF7, and UNC93B1 exert their protective actions during infection.^[^ ^246^, ^247^, ^248^ ^]^ For instance, Hu HL et al.^[^ ^247^ ^]^ find that the subcellular localization of Gadd45b (a host factor) can generate a great influence on the HSV‐1 reactivation state in latently infected primary neurons. When Gadd45b is located in the nucleus of infected neurons, it inhibits the transcription of ICP4 (a viral transcription factor) and the viral late gene expression, thereby suppressing the reactivation of HSV‐1. However, when the virus reactivates, Gadd45b is sequestered in the cytoplasm through an unknown mechanism. These finding emphasizes the antiviral potential of Gadd45b in HSV‐1 infections. In addition, Tucker MH et al.^[^ ^248^ ^]^ discover that the deleterious variants in IRF7 and UNC93B1 may inhibit TLR3‐induced IRF3 transcriptional activity and IRF3‐dependent IFN‐I response, further leading to compromised viral clearance. Therefore, both IRF7 and UNC93B1 are considered antiviral factors. Additionally, the protective role of a microglia subset with high IFN‐I and chemokine expression is also documented.^[^ ^249^ ^]^

β‐herpesvirus

HCMV infections: HCMV is the most common member of the β‐herpesvirus family and is an opportunistic pathogen present in immunodeficient adults. Similar to all herpesviruses, it establishes latency and persists throughout an individual's life. It is reported that infected individuals may experience long‐term adverse effects caused by HCMV‐induced chronic inflammation. Herein, a thorough understanding of the pathogenesis of HCMV infections is crucial for preventing the aforementioned adverse events.^[^ ^250^ ^]^

1) The mechanism of HCMV infections

Monocytes and macrophages are now recognized as crucial cell populations in HCMV infection, with the former allowing for the establishment of latent infection and the latter playing a significant role in productive infection. To elucidate the transcriptional changes of these two cell populations during infection, Schwartz M et al.^[^ ^251^ ^]^ conduct relevant research using single‐cell technology and find that some early viral genes (e.g., genes encoding immediate early proteins IE1 and IE2) play important roles in determining productive infection. The decreased expression of intrinsic interferon‐stimulated genes (ISGs) during the differentiation of monocytes into macrophages may be the reason macrophages are susceptible to productive infection. These findings emphasize that regulating the expression of ISGs may be a potential strategy to control HCMV infection.

2) Host response to HCMV infections

During infection, the proportion of CD4+ naive T cells decreases, while the proportions of CD4+ effector memory T cells and CD45RA+ effector memory T cells increase.^[^ ^252^, ^253^ ^]^ These CD4+ effector memory T cells can differentiate into a subset (CD57+CD27‐CD28‐CD244+CD4+ T cells), characterized by high cytotoxicity and TCR oligoclonality. This subset is capable of controlling HCMV.^[^ ^252^ ^]^ In addition, fibroblastic cells also function in HCMV infections. Pezoldt J et al.^[^ ^254^ ^]^ find that a population of Ly6c1+ red pulp fibroblastic cells can upregulate an ISG‐related transcriptional program and then play a role in innate antiviral defense. These findings underscore the importance of fibroblastic cells in anti‐HCMV immune response.

γ‐herpesvirus infections:

EBV infections: EBV can establish its infection through saliva transition. It can cause infectious mononucleosis and is also closely related to the occurrence of nasopharyngeal cancer and childhood lymphoma. Its first infection target is oral epithelial cells, and then B lymphocytes.^[^ ^255^ ^]^

(1) The pathogenesis of EBV infections

Teplizumab, a FcR non‐binding humanized anti‐CD3 monoclonal antibody, is used to treat type 1 diabetes and can induce partial exhaustion of CD8+ T cells. It is reported that EBV‐seropositive individuals exhibit enhanced response to this drug, which may raise the question of whether EBV can also induce T cell exhaustion. ScRNA‐seq data imply that there are increased regulatory T cells and partially exhausted CD8+ T cells in EBV‐seropositive individuals. And such immune impairments (e.g., increased expression of exhaustion markers in CD8+ T cells) are further enhanced after teplizumab treatment, emphasizing the pathogenic mechanism of EBV by impairing host immunity.^[^ ^256^ ^]^

2) The heterogeneity of host cells during infections

Ziegler P et al.^[^ ^257^ ^]^ use single‐cell sequencing to explore the susceptibility of various epithelial subclusters to EBV in different life cycles and find that lytic infection prefers to appear in suprabasal cells, while latent infection tends to occur in basal/mucosecretory and ciliated cells. For B cells, their fate trajectories and heterogeneous responses in the EBV infection are clearly delineated.^[^ ^255^, ^258^, ^259^, ^260^ ^]^ For instance, primary B cells can form lymphoblastoid cell lines (LCLs) once they are infected. At the early stage of LCL formation, the EBV infection mediates cell proliferation by mimicking the B‐cell activation process, and after the LCL outgrowth, infected B cells exhibit various phenotypes such as activation and plasma cell differentiation.^[^ ^258^ ^]^

3) The host response to EBV infections

Kamga L et al.^[^ ^261^ ^]^ identify the TCRα sequence (KDTDKL) shared by all included subjects and reveal conserved residues in this sequence (a lysine at position 1) that might be responsible for viral recognition. These findings deepen our understanding of how the TCR repertoire exerts its protective role against EBV.

#### 3.3.4 Influenza A

Influenza A virus (IAV) is the primary pathogen responsible for influenza, that can cause pandemic public health concerns.

1) The heterogeneous behaviors of IAV

The behaviors of IAV viruses are heterogeneous among different host cells.^[^ ^262^, ^263^, ^264^, ^265^, ^266^, ^267^ ^]^ For instance, there are significant differences in the transcription efficiency of IAV among various host cells. In some host cells, viral transcripts can account for half of the total mRNA content, while in other cells, this proportion may be less than one percent. The low abundance of viral transcripts may be due to the lack of expression of any of the four genes encoding viral ribonucleoproteins (RNPs) in host cells, or it may be due to the stochastic loss of viral RNPs after infection but before transcription initiation.^[^ ^262^ ^]^

While Russell AB et al.^[^ ^266^ ^]^ propose that the diversity of defects may also be a potential cause of the above differences based on scRNA‐seq. The diversity and heterogeneity of defective viral genomes (DVGs) have also been reported in another single‐cell sequencing‐related study.^[^ ^265^ ^]^ This study further points out that the accumulation of DVG ultimately affects viral replication by competing with full‐length viral genomes and also regulates host immune responses through the activation of the IFN signaling cascade.^[^ ^265^ ^]^

2) The immune profiles and antiviral response of influenza A patients

Host cells also exhibit varied responses, which are manifested by increased diversity of memory B cells,^[^ ^268^ ^]^ accumulated lung‐resident memory CD8+ T cells,^[^ ^269^ ^]^ expanded CD38+HLA‐DR+PD‐1+CD8+ T cells,^[^ ^270^ ^]^ and others.^[^ ^270^, ^271^, ^272^, ^273^, ^274^, ^275^, ^276^, ^277^, ^278^, ^279^ ^]^ These cells (e.g., M2e‐specific lung resident T helper type 17 and IgA‐expressing cells in the respiratory mucosa), and some molecules (e.g., IFN signaling‐related molecules and colony‐stimulating factor) within them, are crucial for the host's immune response to IAV.^[^ ^280^, ^281^, ^282^ ^]^ For example, group 2 innate lymphoid cells (ILC2s) play a positive role in reducing inflammation and repairing tissues. At the single‐cell level, a transcriptionally distinct subset of ILC2s has been identified.^[^ ^276^ ^]^ This subset exhibits a high expression of BATF (a transcription factor). BATF can bind to the cis‐regulatory elements of wound healing genes, modulate their chromatin accessibility, and further promote their expression in these cells, endowing them with functions that alleviate inflammation and promote wound healing. Therefore, BATF is crucial for maintaining ILC2s’ function and may serve as a target for alleviating severe influenza‐related inflammation. At the molecular level, the importance of IFN signaling has been repeatedly emphasized.^[^ ^31^, ^283^, ^284^, ^285^, ^286^, ^287^, ^288^ ^]^ For instance, during infection, upregulated lncRNA‐GM inhibits GSTM1‐TBK1 interaction by directly binding to GSTM1, thereby interfering with GSTM1‐mediated S‐glutathione of TBK1. This alternation further promotes IFN‐I production and enhances antiviral response.^[^ ^288^ ^]^

Other concerns, such as IAV‐others virus coinfection and the influence of cigarette smoke during influenza, are also documented.^[^ ^289^, ^290^, ^291^, ^292^, ^293^, ^294^, ^295^, ^296^ ^]^

### 3.4 Bacterial Infections

In this section, we primarily focus on TB and bacterial sepsis. Evidence about the other 14 bacterial infections is shown in **Table** **3**.

**Table 3:** Evidence related to other bacterial infections.

Infection or disease	Sc^a)^‐technology	Sample type	Main finding	Refs.
Bacteria‐induced carious lesions	ScRNA‐seq^b)^	Human pulp tissues	1) The cellular profiling of carious teeth is provided (e.g., an increased fibroblast subset expressing ECM^c)^‐related molecules). 2) In carious pulp tissues, active immune response is accompanied by alterations in fibroblast and mesenchymal stem cell clusters. These alternations include increased ECM components‐encoded genes (e.g., COL1A1^d)^) and an accumulated fibroblast subset with myofibroblasts.	[297]
*Chlamydia trachomatis* infections	ScRNA‐seq	HEp2^e)^ epithelial cell monolayers	1) The specific cellular trajectory is described. And the expression patterns of metallothioneins and genes related to cell cycle, innate immune responses, cytoskeletal components, lipid biosynthesis, and cellular stress are also provided.	[298]
*Cutibacterium acnes*‐induced acne	ScRNA‐seq	Human and mouse skin tissues	1) There is the colocalization of PREF1^f)^ (a biomarker of adipogenesis) and cathelicidin (an antimicrobial peptide) in perifollicular dermal preadipocytes of skin lesions. 2) *Cutibacterium acnes* promotes the production of cathelicidin in preadipocytes in a TLR ^g)^2‐dependent way. 3) Retinoic acid can inhibit the ability of *Cutibacterium acnes* to form acne‐like lesions and adipogenesis, and improve the expression of cathelicidin in preadipocytes.	[299]
	ScRNA‐seq and spatial transcriptomic sequencing	Human skin tissues	1) GRN^h)^ increases the expression of proinflammatory cytokines and chemokines (e.g., Il^i)^‐18, CCL^j)^5, and CXCL^k)^2) in TREM^l)^2 macrophages. 2) IL‐13 induced‐IL‐13RA1 activation in HaCaT^m)^ cells promote the dysregulation of genes related to hyperkeratinization, such as KRT^n)^17 and KRT16.	[300]
	ScRNA‐seq	Human skin tissues	1) There is increased glycolytic activity in acne lesions, indicating a Warburg‐like effect that promotes inflammation.	[301]
*Desulfobulbus* and *Desulfovibrio*‐related periodontitis	ScDNA‐seq	Human subgingival and teeth paper point samples and curette samples	1) The genomic and functional atlas of these *Deltaproteobacteria* in the oral environment is provided. 2) Genes related to adhesion, stress resistance, defense, and host‐cell interactions, and degradation are crucial for a host‐adapted lifestyle. The virulent characteristics of these organisms contribute to the relationship between organisms and periodontitis.	[302]
*Escherichia coli* induced‐liver abscesses	ScRNA‐seq	Mouse liver CD45+ cells	1) The heterogenous immune cells (macrophages, neutrophils, etc.) near necrotic regions are related to liver abscesses. 2) There is inhibited activation of early inflammatory responses. 3) TLR4 signaling plays a role in the balance between liver abscess formation and bacterial elimination.	[303]
*Escherichia coli* infections	ScTCR‐seq^o)^	Mouse thymus CD4+CD8+ T cells	1) There is a relationship between increased TCR^p)^β transcripts and *Escherichia coli* infection. 2) During *Escherichia coli* infection, some Vβ genes (Vβ12‐1, 12‐2, 13‐1, and 13‐2, named 12–13) is well expressed. The expression frequency of the Vα gene located in the distal region of the TCRα site is increased in the Escherichia coli infection group.	[304]
*Helicobacter pylori* infections	ScRNA‐seq and CyTOF^q)^	Human gastric tissues	1) *Helicobacter pylori* preferentially bind to the most differentiated pit cells marked by high expression of GKN^r)^1, GKN2, and PSCA^s)^. Such a preference is associated with cell size. 2) The preferential attachment to large cells of *Helicobacter pylori* is related to urea chemotaxis.	[305]
	ScRNA‐seq	Human gastric mononuclear cells	1) ILC ^t)^3s are the main cell type of human gastric mucosa, but not ILC2s. 2) In the gastric mucosa from infected individuals, the ratio of NK^u)^ p44+ ILC3s in total ILCs is raised. And the abundance of CD11c+ myeloid cells, activated CD4+ T cells, and activated B cells increase. 3) In infected individuals, B cells are induced into enter an activated state and progress to a highly proliferating germinal‐center stage and plasmablast maturation. Such processes are related to the presence of tertiary lymphoid structures in the gastric lamina propria.	[306]
	ScRNA‐seq and scTCR‐seq	Human PBMC^v)^ and gastric biopsies	1) The human gastric ecosystem undergoes impaired antigen presentation and phagocytosis function after infection. 2) *Helicobacter pylori* can drive the differentiation from monocytes to C1QC^w)^+ macrophages. And T cell responses show broad functional impairment and hyporesponsiveness with restricted clonal expansion capacity. 3) An HLA^x)^‐DRs‐ and CTLA4‐expressing T cell subset in *Helicobacter pylori*‐inhabited stomach may be related to immune evasion.	[307]
	ScRNA‐seq	Human and mouse gastric *Helicobacter pylori*‐specific tissue‐resident memory T cells	1) The transcription factor, Hobit, controls the induction and development of gastric tissue‐resident memory T cells. And the Hobit expression is regulated by the presence of the *Helicobacter pylori* virulence factor Cytotoxin‐associated gene A. 2) *Helicobacter pylori*‐specific CD4+ and CD8+ tissue‐resident memory T cells reside in the stomach for a long time and provide complete protection against reinfection with the help of neutrophils. 3) Gastric CD8+ tissue‐resident memory T cells show different Hobit expression levels, and there is certain heterogeneity within these cells.	[308]
Lepromatous leprosy	ScRNA‐seq	Human skin biopsies and PBMC	1) In skin lesions, increased APOE^y)^ in macrophages featured by a high level of LIPA^z)^ shows the negative relationship with HLA‐DQB2 and MIF that encode a pro‐inflammatory and anti‐microbial cytokine. Exhausted CD8+ T cells are characterized by augmented TIGIT^aa)^ and LAG3^ab)^. And inhibitory immune receptors‐induced interactions between skin immune cells that are enhanced. 2) In PBMCs, HLA‐DR^high^FBP1^ac)^ ^high^ monocytes upregulate the expression of APOE. There is a potential relationship between expanded regulatory T cells and lepromatous leprosy.	[309]
Non‐tuberculous mycobacterial infections	ScRNA‐seq	Human whole blood	1) Patients with non‐tuberculous mycobacterial pulmonary disease have high cytotoxic T cells and increased abundance of inflammatory and activated mononuclear phagocytes subclusters. 2) Specific expansion of IFIT^ad)^1+ neutrophil subsets and the CXCL8‐CXCR1/2 axis may be related to the pathogenesis of non‐tuberculous mycobacterial pulmonary disease.	[310]
Non‐tuberculous mycobacterial infections	ScRNA‐seq	Rhesus macaques bronchoalveolar lavage	1) The immune alterations of the lungs are described, such as decreased alveolar macrophages in 8 days post‐infection. 2) CD8+ T cells show high cytotoxicity over the course of the infection in both young and aged animals. 3) There is a heightened inflammatory response in alveolar macrophages from aged animals.	[311]
*Porphyromonas gingivalis* and *Enterococcus faecalis* infections	ScRNA‐seq	Human dental pulp stem cells	1) In the *Porphyromonas gingivalis* infection, a cell subset with high expression of THBS1^ae)^ and PTGS2^af)^ is enriched. The expression of genes related to matrix formation and mineralization (e.g., THBS1) and the cellular response to hypoxia (e.g., HILPDA^ag)^) changes. TGF‐β^ah)^/SMAD^ai)^, NF‐κB^aj)^ and MAPK/ERK^ak)^ signaling pathways act against *porphyromonas gingivalis* infection. And *Porphyromonas gingivalis* induces a hypoxia state to regulate the differentiation of human dental pulp stem cells. 2) In the *Enterococcus faecalis* infection, a cell subset with similar characteristics of myofibroblasts significantly expresses ACTA2^al)^. Also, CCL2 that is related to leukocyte chemotaxis is highly expressed. *Enterococcus faecalis* mediates the differentiation of human dental pulp stem cells into fibroblast‐like cells.	[312]
*Propionibacterium acnes* infections	ScRNA‐seq and CyTOF	Mouse skin cDC1s^am)^	1) cDC1s, but not cDC2s, participate to *Propionibacterium acnes*‐induced immune responses by regulating the recruitment, survival, and function of neutrophils. Such action is dependent on the secretion of VEGF^an)^‐α by activated EpCAM+CD59+Ly‐6D+ cDC1s. 2) Dermal cDC1s‐induced neutrophil recruitment is also found in other infections.	[313]
*Salmonella* infections	ScRNA‐seq	Mouse bone marrow derived macrophages	1) Only a fraction of *Salmonella* underwent PhoP‐mediated lipopolysaccharides modifications, and modified lipopolysaccharides can drive the type I interferon response of host macrophages.	[314]
	ScRNA‐seq	Mouse bone marrow derived macrophages	1) Macrophages containing non‐growing *Salmonella* are in proinflammatory M1 polarization state, while these harboring growing *Salmonella* are in anti‐inflammatory and M2‐like state.	[315]
	ScRNA‐seq	Human PBMC	1) Infected cells primarily altered genes related to defense response to virus, type I interferon signaling pathway, and inflammatory response.	[316]
*Staphylococcus aureus‐*induced sepsis	ScRNA‐seq	Mouse renal CD45+CD3+CD4+YFP+ cells	1) Th^ao)^17 fate cells can product Th17 and Th1 cytokines and one subset of these cells shows the Th1 expression profile. 2) IL‐17A but not interferon‐γ is crucial for bacterial control. The Th17‐Th1 transdifferentiation in Th17 fate cells contributes to increased bacterial loads.	[317]
*Staphylococcus aureus*‐induced craniotomy infections	ScRNA‐seq	Mouse CD45+ cells from the brain, galea, bone flap and blood	1) The transcriptional heterogeneity of resident microglia and infiltrating monocytes in the brain, as well as granulocytes in the subcutaneous galea and bone flap, is described. 2) In brain, microglia undergo a phenotypic transition from homeostatic/anti‐inflammatory to proinflammatory and proliferative state. While granulocytes undergo a phenotype ranging from granulocyte myeloid‐derived suppressor cells to mature neutrophils. 3) In the galea, interferon signaling‐ and cell cycle‐related genes are enriched in granulocyte myeloid‐derived suppressor cell‐like populations and neutrophils. 4) In the galea and bone flap, decreased polymorphonuclear cells and granulocyte myeloid‐derived suppressor cells are related to increased bacterial burden.	[318]
*Staphylococcus aureus*‐induced cellulitis	ScRNA‐seq	Mouse CD45+ cells from brain and galea	1) Reactive oxygen species/reactive nitrogen species‐, lysosome‐, iron uptake‐, and transport‐related pathways are enriched in phagocytic cells, while nonphagocytic cells are characterized by enhancive ribosomal, interferon, and hypoxia signatures. 2) NOX^ap)^2 can inhibit *S. aureus* burden, leukocyte recruitment, and intracellular bacterial load.	[319]
	ScRNA‐seq and ScATAC‐seq	Mouse skin tissues	1) Hypodermal macrophages are found to be able to clear hyaluronic acid that offers protection against *Staphylococcus aureus*. Such ability is dependent on LYVE^aq)^‐1 expression that is regulated by IGF^ar)^1, implying the potential of Hypodermal macrophages and IGF1 as novel therapeutic targets.	[320]
Implant‐related *Staphylococcus aureus* infections	ScRNA‐seq	Human bone‐implant interfaces	1) There is a bipolar differentiation pattern of fibroblasts, and forskolin may act as a differentiation regulator. 2) Fibroblasts in periprosthetic tissues of *Staphylococcus aureus*‐infected periprosthetic joint tend to enter a pro‐inflammatory state by activating NPAS ^as)^ 2and TFEC^at)^.	[321]
*Staphylococcus aureus* infections	ScRNA‐seq	Mouse liver tissues	1) BIRC^au)^5 from CX3Cr^av)^1‐expressing cells can inhibit the survival of *Staphylococcus aureus*.	[322]
	ScRNA‐seq	Mouse keratinocytes	1) Keratinocytes produce IL‐24 after *Staphylococcus aureus* infection.	[323]
	ScRNA‐seq and single cell V(D)J sequencing.	Human PBMC	1) *Staphylococcus aureus*‐specific mAb BC153 targets wall teichoic acid, and cross‐reactive mAbs BC019, BC020, and BC021 target lipoteichoic acid. 2) All these mAbs can induce Fc‐dependent phagocytosis of *staphylococci* by neutrophils.	[324]
*Staphylococcus aureus*‐related cutaneous inflammation	ScRNA‐seq	Human skin keratinocytes	1) *Staphylococcus aureus* specifically promotes the expression of genes related to epidermal inflammation and antimicrobial response in a spinous cell subset. 2) Basal proliferating cells are induced by *Staphylococcus aureus* to specifically express IL‐1α and IL‐1β, suggesting the release of pro‐inflammatory activation signals to other cutaneous subsets.	[325]
*Staphylococcus aureus*‐related atopic dermatitis	ScRNA‐seq	Mouse skin tissues	1) Under Th2 inflammatory conditions, reduced antimicrobial peptides caused by IL‐4Rα activation allow *Staphylococcus aureus* to survive on the skin.	[326]
*Streptococcus pneumoniae* infections	ScRNA‐seq	Mouse AT2^aw)^ cells	1) The transcriptome of proliferative AT2 cells is regulated by activating ATF^ax)^3 and THRA transcription factors. 2) Overexpressed ATF3 and THRA^ay)^ transcription factors drive the proliferation of AT2 cells and promote lung function after injury.	[327]
X‐linked severe combined immunodeficiency related‐various infections	ScRNA‐seq	Human bone marrow mononuclear cells	1) In both maternal and autologous T and natural killer T cells, the expression of some important cytokines (e.g., GZMB^az)^) against infections increases, while the expression of some transcription factors (e.g., FOS^ba)^) related to lymphocyte activation, proliferation, and differentiation decreases. And many inhibitory factors (e.g., LAG3) showed augmented expression.	[328]

^a)^ single‐cell;

^b)^ single‐cell RNA sequencing;

^c)^ extracellular matrix;

^d)^ collagen type I alpha 1;

^e)^ Human Epithelioma‐2;

^f)^ preadipocyte factor 1;

^g)^ Toll‐like receptors;

^h)^ granulin;

^i)^ Interleukin;

^j)^ chemokine (C‐C motif) ligand;

^k)^ (C‐X‐C motif) ligand;

^l)^ The triggering receptors expressed on myeloid cells;

^m)^ human keratinocytes;

^n)^ keratin;

^o)^ single‐cell T cell receptor sequencing;

^p)^ T cell receptor;

^q)^ cytometry by time of flight;

^r)^ gastrokine;

^s)^ prostate stem cell antigen;

^t)^ Innate lymphoid cells;

^u)^ natural kill;

^v)^ peripheral blood mononuclear cell;

^w)^ complement C1q subcomponent subunit C;

^x)^ human leukocyte antigen;

^y)^ apolipoprotein E;

^z)^ lipase A;

^aa)^ T cell immunoreceptor with immunoglobulin and ITIM domain;

^ab)^ lymphocyte‐activation gene 3;

^ac)^ fructose‐1,6‐bisphosphatase;

^ad)^ Interferon‐induced tetrapeptide repeat;

^ae)^ thrombospondin‐1;

^af)^ prostaglandin‐endoperoxide synthase 2;

^ag)^ hypoxia‐inducible lipid droplet‐associated protein;

^ah)^ transforming growth factor;

^ai)^ small mother against decapentaplegic;

^aj)^ nuclear factor kappaB;

^ak)^ mitogen‐activated protein kinase /extracellular signal‐regulated kinase;

^al)^ actin alpha 2;

^am)^ type 1 conventional dendritic cells;

^an)^ vascular endothelial growth factor;

^ao)^ T helper cells;

^ap)^ NADPH oxidases 2;

^aq)^ lymphatic vessel endothelial hyaluronan receptor;

^ar)^ insulin‐like growth factor;

^as)^ neuronal PAS domain protein;

^at)^ transcription factor EC;

^au)^ baculoviral IAP repeat‐containing;

^av)^ CX3C motif chemokine receptor;

^aw)^ alveolar type 2;

^ax)^ activating transcription factor;

^ay)^ thyroid hormone receptor alpha;

^az)^ granzyme B;

^ba)^ c‐fos protein.

#### 3.4.1 TB

TB is a representative bacterial infectious disease, generating a serious global health catastrophe. *Mycobacterium tuberculosis* (MTB), the pathogen of TB, enters the host through the respiratory tract and then interacts with the airway epithelial cells and phagocytic cells. Some hosts can control MTB to maintain an inactive state, clinically defined as latent TB individuals. However, these individuals develop active TB patients after several months or even decades. Certainly, some hosts who fail to control MTB may directly become active TB patients once infected. Such phenotypic differences highlight the need to explore TB at a finer level. Currently, some focuses including granuloma microenvironments^[^ ^329^, ^330^ ^]^ and the behaviors of various cell subsets^[^ ^54^, ^331^, ^332^, ^333^, ^334^, ^335^, ^336^, ^337^, ^338^, ^339^ ^]^ are discussed at the single‐cell level, as well as the roles of some specific processes (e.g., cell death),^[^ ^340^, ^341^ ^]^ *Bacillus Calmette‐Guérin*‐induced immunity^[^ ^342^, ^343^ ^]^ and the relationships of TB and other infections.^[^ ^344^, ^345^, ^346^, ^347^ ^]^

1) Heterogeneity of granuloma and host cells in TB

Granuloma is essentially a dense and organized aggregate of mature macrophages in response to persistent stimulation, as well as a result of local interactions between host‐MTB at the site of infection. The granuloma microenvironment determines TB onset and development to a certain extent. Single‐cell sequencing has advantages for studying the multicellular environment of granulomas. Lienard J et al.^[^ ^329^ ^]^ illustrate that ESAT‐6 secretion system‐1 (ESX‐1), the major MTB virulence determinant, mediates immunopathology by inducing neutrophil accumulation in granulomas, while monocytes play an antagonistic role through the iNOS‐dependent pathway. And it is also reported that MTB can achieve infection and survival by recruiting and influencing the differentiation of mononuclear phagocytes through ESX‐1.^[^ ^348^ ^]^ Gideon HP et al.^[^ ^330^ ^]^ focus on the influencing factors of MTB persistence and growth in granulomas. The high MTB burden granulomas are characterized by mast cells, endothelial cells, fibroblasts, and plasma cells, while in low MTB burden granulomas, type 1‐type 17, stem‐like, and cytotoxic T cells are enriched. Granuloma heterogeneity indicates the importance of cellular ecosystems and emphasizes the value of host‐derived molecules in targeted anti‐TB therapy.

T cells are also major targets that MTB interacts and their extensive transcriptome and cell receptor programming in TB are described.^[^ ^335^, ^338^, ^339^, ^349^, ^350^, ^351^, ^352^, ^353^, ^354^, ^355^, ^356^, ^357^, ^358^, ^359^, ^360^, ^361^ ^]^ Further, gene biomarkers of exhausted T cells (CD4+ T cells: H1FX, ZFP36L2, VIM, and PPP1R15A; CD8+ T cells: ITM2C),^[^ ^335^ ^]^ and 24 TCR similarity groups correlated to TB control^[^ ^338^ ^]^ are summarized. Besides, some new T cell subpopulations and their roles are documented. For instance, a novel cluster, named CD8+ γδ T cells, is identified in patients with chronic MTB infection, and these cells are hyporesponsive to TCR‐mediated signaling but can generate strong CD16‐mediated cytolytic responses.^[^ ^336^ ^]^ And CD16‐mediated cytolytic response is one of the major ways NK cells function and is critical for controlling MTB infection, suggesting that CD8+ γδ T cells may exert their protective action in TB‐like NK cells. Besides, polyfunctional CD4+ T cells, characterized by CD1‐restriction, T helper type 1 (Th1) response, and cytotoxicity, are also reported to play a protective role in TB.^[^ ^349^ ^]^

2) Mechanism of the persistent MTB infection

Srinivas V et al.^[^ ^331^ ^]^ and Pisu D et al.^[^ ^332^ ^]^ devote to using single cell technology to answer the question of factors influencing MTB persistence and growth, while they choose macrophages, the main MTB residence, as the research object. The former demonstrates the mechanism by which MTB persists, including upregulation of vapC30, mazF, and relA/spot. And host cells with persistent MTB are characterized by a low‐oxygen metabolic state, underscoring the value of activating respiration in eliminating MTB.^[^ ^331^ ^]^ The latter identifies three distinct monocyte‐derived interstitial macrophage subpopulations with different MTB phenotypes, as well as pro‐inflammatory alveolar macrophages related to stressed MTB.^[^ ^332^ ^]^ Moreover, they find that chromatin accessibility of some inflammatory genes (IL‐1b, IL‐6, Fos, etc.) in these cells is significantly altered, signifying the regulatory role of epigenetics in the heterogeneous macrophage responses. Such TB‐driven heterogeneous reactions of macrophages are also observed by others.^[^ ^54^, ^333^, ^350^, ^361^, ^362^, ^363^, ^364^ ^]^

3) TB‐related biomarkers

NK cells, especially CD27+ NK cells, are significantly expanded in the lung tissues of latent TB infection, while plasmacytoid DCs, IFN‐responsive macrophages, and activated T cells are evidently accumulated in pulmonary TB rhesus macaques.^[^ ^365^ ^]^ These cell subsets may serve as features of different TB states. In peripheral blood mononuclear cells (PBMCs), some NK cell subpopulations (e.g., CD3‐CD7+GZMB+ NK cells) and some T cell subsets (e.g., CD154+CD4+ T cells) also has a potential of being a biomarker for different TB states.^[^ ^366^, ^367^, ^368^ ^]^ In bronchoalveolar lavage fluid, monocyte macrophage‐CCL23, ‐FCN1, and ‐SPP1 exhibit strong association with TB states, and in particular, monocyte macrophage‐CCL23 is regarded as a biomarker.^[^ ^369^ ^]^ Jiang J et al.^[^ ^337^ ^]^ report a series of PBMC‐derived cell subpopulations (GZMK‐expressing CD8+CD161‐Ki‐67‐ and CD8+Ki‐67+ T cells, etc.) that are associated with the extent of TB lesions. Besides, GZMK‐expressing CD8+ T cells in pleural fluid can be considered as a feature of tuberculous pleural effusion,^[^ ^370^ ^]^ and a macrophage‐RGS1^high^ subset in tissues can work as a biomarker for the diagnosis and treatment efficacy monitoring of patients with osteoarticular tuberculosis.^[^ ^371^ ^]^ Some gene biomarkers (e.g., ACSL4, CTSB, and TLR4) are also documented.^[^ ^372^ ^]^

**Figure 3:**
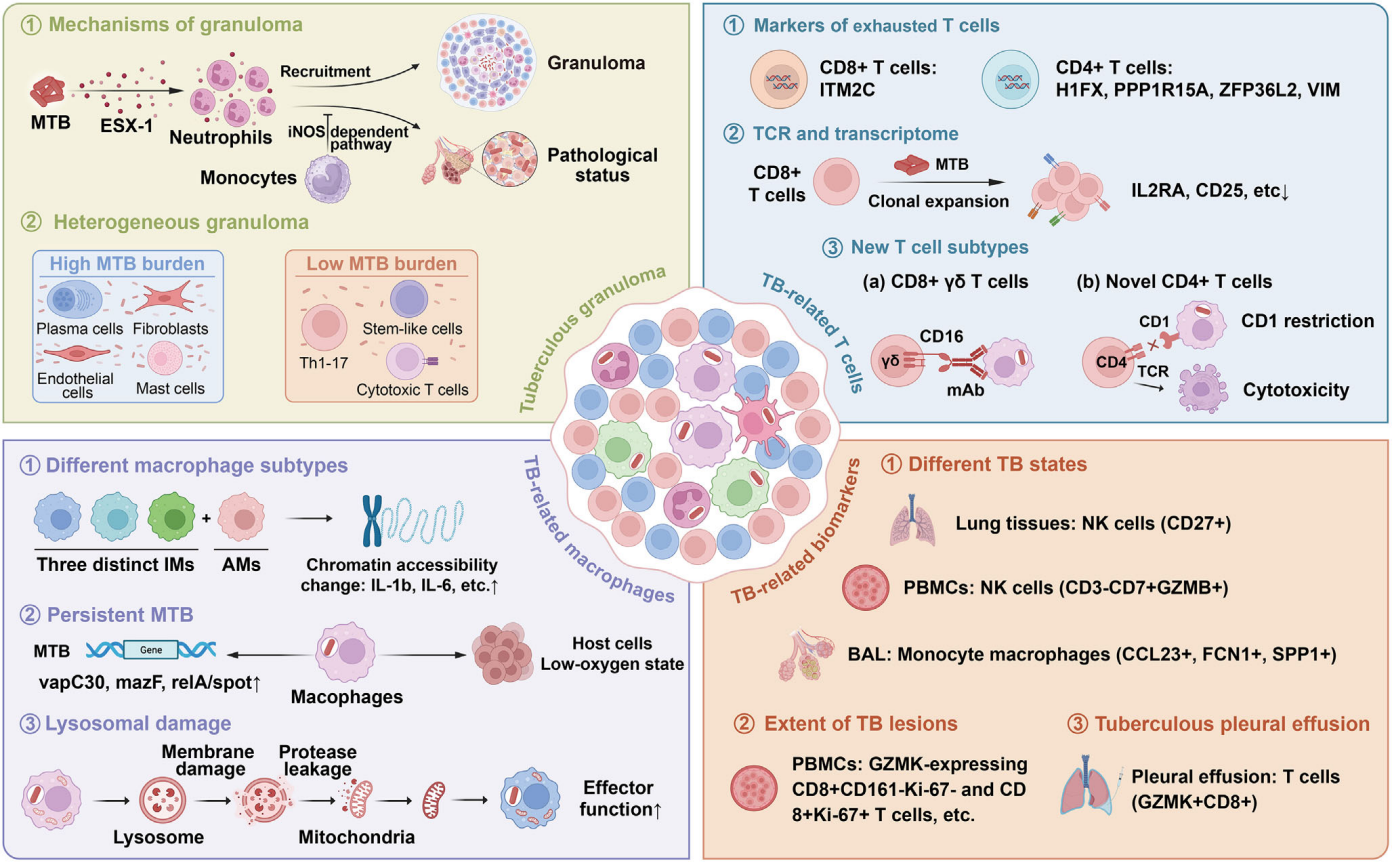
The characteristics of tuberculosis infection at the single‐cell level. Features and some potential mechanisms of granulomatous microenvironments, macrophages, and T cells were visualized. Some tuberculosis‐related biomarkers were summarized. (Created with BioRender.com). Abbreviation: TB: tuberculosis; MTB: *Mycobacterium tuberculosis*; ESX‐1: ESAT‐6 secretion system‐1; iNOS: inducible nitric oxide synthase; Th1: T helper type 1; IM:interstitial macrophage; AM: alveolar macrophage; mAb: monoclonal antibody; TCR: T cell receptor; NK: natural killer; PBMCs: peripheral blood mononuclear cells; BAL: bronchoalveolar lavage.

TB‐related evidence is shown in **Figure** **3**.

#### 3.4.2 Bacterial Sepsis

Sepsis is a life‐threatening organ dysfunction syndrome, resulting in a high mortality rate worldwide.^[^ ^373^ ^]^ Sepsis cannot be simplified as a process of systemic inflammation response or immune dysregulation, but rather involves the altered function of multiple organs in the body. Imbalanced inflammatory responses, immunosuppress, abnormal neuroendocrine‐immune network, mitochondrial damage, coagulation dysfunction, endoplasmic reticulum stress, autophagy, and other processes are considered potential mechanisms underlying sepsis.^[^ ^374^ ^]^

1) The immune profile and pathogenesis of sepsis

During sepsis, the proportions of hematopoietic stem cells, myeloid cells, and T cells significantly decrease, while neutrophils expand remarkably.^[^ ^375^, ^376^, ^377^, ^378^, ^379^ ^]^ Transcriptionally, some myeloid subpopulations exhibit high expression of damage‐associated molecular pattern related‐ and IFN‐γ signaling related‐genes (e.g., S100A8 and ISG15).^[^ ^375^ ^]^ Specifically, CD52 in lymphocytes is significantly elevated, showing a potential association with improved prognosis in sepsis patients. Therefore, CD52 could be considered as a prognostic marker or therapeutic target.^[^ ^376^ ^]^

In terms of pathogenesis, immunosuppress is a typical feature of sepsis, which can be manifested as cell exhaustion or dysfunction and lymphopenia.^[^ ^380^ ^]^ It has been documented that many cells including monocytes/macrophages and T cells suffer from exhaustion during sepsis,^[^ ^381^, ^382^ ^]^ and mechanically, blood T cell exhaustion may be triggered by monocytes in an IL‐1B signal pathway‐dependent manner.^[^ ^382^ ^]^ And sepsis‐related liver immune dysfunction can be attributed to increased lymphocyte apoptosis and abnormal neutrophil recruitment.^[^ ^383^ ^]^ For lymphopenia, Lai Y et al.^[^ ^384^ ^]^ pinpoint that PRMT4 is significantly augmented in activated T cells, inducing lymphocyte apoptosis by caspase 3‐mediated cell death signaling.^[^ ^384^ ^]^ These results underline the potential of PRMT4 as a therapeutic target for sepsis.

### 3.5 Fungal Infections

Research on fungal infections at the single‐cell level is limited, with only one study available for discussion in the following section.

#### 3.5.1 Cryptococcus Neoformans (C. Neoformans) Infections

C. neoformans is an opportunistic pathogen that causes a life‐threatening cryptococcal meningitis. Since they are ubiquitous in the environment, it is easy for humans to inhale their spores or yeast cells into the lungs.^[^ ^385^ ^]^ Typically, C. neoformans is either removed or goes dormant, while in immunocompromised hosts, C. neoformans can replicate in the lungs and disseminate into the blood, causing pneumonia, meningitis, and other diseases.

1) Heterogeneity of host cell response to infection

Antigen‐presenting cells (APCs) such as DCs and tissue resident macrophages function as the first line of host defense against C. neoformans.^[^ ^386^ ^]^ However, distinct subclusters of APCs exhibit different behaviors during C. neoformans infection, which may result in diverse disease progression. Nelson BN et al.^[^ ^387^ ^]^ explore the responses of different phagocytic APCs to C. neoformans based on scRNA‐seq. Although all analyzed subclusters can interact with C. neoformans, they differ in their killing capabilities. TNF‐α is related to C. neoformans interactions and uptake, and it is reported that the level of TNF‐α in innate immune cells is significantly augmented. Besides, an increase in FABP4 promotes fatty acid metabolism, which is a characteristic of cells with strong cryptococcal killing ability.^[^ ^387^ ^]^ These findings indicate the important features of cells with varied killing abilities and thus provide evidence for the development of therapeutic targets.

### 3.6 Parasitic Infections

For the parasitic infection section, our focus is on malaria. The other 8 parasitic infections are summarized in **Table** **4**.

**Table 4:** Evidence related to other parasitic infections.

Infection or disease	Sc^a)^‐technology	Sample type	Main finding	Refs.
*Cryptosporidium* infections	ScRNA‐seq^b)^	Mouse epithelial layer	1) There are increased young cells and decreased mature cells in infected intestines. 2) The apoptosis of infected cells was lower than that of bystander cells	[388]
*Echinococcus multilocularis* infections	ScRNA‐seq and ScTCR‐seq^c)^	Human liver tissues	1) The expression of CD244 in CD8+ T cells of the patient's liver tissue increases. which contributes to CD8+ T cell exhaustion. 2) CD244+CD8+ T cells show a more terminal differentiation phenotype, characterized by decreased secretion of the inflammatory cytokines.	[389]
*Encephalitozoon intestinalis* infections	ScRNA‐seq	Primary human monocyte‐derived macrophages and *Encephalitozoon intestinalis*	1) The parasite undergoes extensive transcriptional changes throughout the life cycle. 2) Only a small portion of the infected macrophages respond, while transcription remains unchanged in most cells, suggesting that the majority of parasites can escape to host surveillance.	[390]
*Leishmania major* infections	ScRNA‐seq	Mouse infected ear tissues	1) Genes related to IFN^d)^‐induced GTPases and antigen presentation are upregulated in macrophages, resident macrophages and inflammatory monocytes during infection. 2) The antigen presentation pathway is increased during infection, while eIF4/p70S6k, mTOR^e)^ and EIF^f)^ 2 signaling pathways are significantly inhibited in various cells (e.g., blood and lymphatic endothelial cells).	[391]
	ScRNA‐seq	Mouse infected ear tissues	1) Coordinated lysosomal expression and regulation signaling with increased cathepsin and H^+^‐ATPase transcripts are increased during infection. 2) Infected cells have downregulated EIF2 signaling including decreased small and large ribosomal subunit (Rps^g)^ and Rpl^h)^) genes in infected dermal macrophages. 3) Bystander dermal macrophages show inhibited EIF2 signaling, including EIF, Rps, and Rpl transcripts compared with these from normal skin.	[392]
Leishmania promastigotes	ScDNA‐seq^i)^	Promastigotes	1) Aneuploidy changes arise from the polyclonal selection of pre‐existing karyotypes, coupled with rapid de novo changes in chromosome copy numbers throughout evolution. 2) In the case of miltefosine, initial parasite adaptation is related to independent point mutations in a miltefosine transporter gene, whereas aneuploidy changes appear later, following exposure to elevated drug concentrations.	[393]
*Nippostrongylus brasiliensis* infections	ScRNA‐seq	Mouse intestinal tissues	1) The depletion of SOX4^j)^ impairs the tuft cell hyperplasia, the function of intestinal stem cells and secretory differentiation, and delays parasite clearance. 2) SOX4 can trigger the differentiation of tuft and enteroendocrine cells in an Atoh1‐independent way.	[394]
*Pneumocystis* pneumonia	ScRNA‐seq and ScTCR‐seq	Mouse lung tissues	1) Clonal cells are predominantly consisted of CD4+ T cells responding to Pneumocystis, featured by high expression of T cell activation‐related genes. There is decreased TCR^k)^ diversity in CD4+ T cells, but increased diversity in CD8+ T cells in the infected group. 2) The majority of T helper type 17 cells are clonal CD4+ T cells, displaying a tissue‐resident memory‐like T helper type 17 cell phenotype. 3) The infected group displays preferential usage of TCRβ VDJ^l)^ genes.	[395]
*Toxoplasma gondii* infections	ScRNA‐seq	Mouse spleen CD4+CD25+ regulatory T cells	1) Transfer of pathogen‐activated maternal regulatory T cells can rescue the maternal immune activation‐induced abnormalities. 2) A unique group of pathogen‐activated regulatory T cells among pathogen‐activated maternal regulatory T cells is identified. Differentially expressed genes in these cells are related to leukocyte cell‐cell adhesion, proliferation, T cell activation, chemotaxis, migration, cytokine production, and tolerance induction. 3) These regulatory T cells can infiltrate into the brain parenchyma.	[396]
	ScRNA‐seq	Human foreskin fibroblasts	1) C‐Myc^m)^ and NF‐κB^n)^ signaling and energy metabolic pathways are upregulated during infection. 2) Cells with low parasite load exhibit enhanced IFN‐I and ‐II response pathways, while such upregulation is not observed in infected cells. 3) Differentiation states of parasites lead to the differential expression of BAG1^o)^ and SAG1^p)^ in host cells. 4) In cells containing *Toxoplasma gondii* ^BAG1+/SAG1−^, NF‐κB, inflammatory response pathways, and IFN‐γ response pathways are downregulated, whereas this downregulation effect is reversed in cells containing *Toxoplasma gondii* ^BAG1‐/SAG1+^.	[397]
	ScRNA‐seq	*Toxoplasma gondii* from human foreskin fibroblasts	1) BFD1^q)^ is necessary for differentiation of *Toxoplasma gondii*, and can bind to the promoters of numerous stage‐specific genes.	[398]
	ScRNA‐Seq	Mouse BMDCs^r)^	1) BMDCs exhibit varied responses to *Toxoplasma gondii* infection, and two parasite lineages distinctly control subpopulations of infected BMDCs. 2) GRA^s)^45 is increased in *Toxoplasma gondii* PTG^t)^, which shows co‐expression with genes related to innate immune response, inflammation and autophagy. While increased genes in *Toxoplasma gondii* LDM^u)^ are co‐expressed with those associated with filamentation and pathogenesis.	[399]
	ScRNA‑seq	Human blood monocytes and *Toxoplasma in monocytes*	1) Toxoplasma‐exposed and unexposed monocytes can be distinguished by a CD14+CD16‐ monocyte subset at the transcriptome level. 2) Infection‐distinguishing monocytes secrete elevated chemokines (e.g., CCL2^v)^ and CXCL5^w)^).	[32]
*Toxoplasma gondii* infections	ScRNA‑seq and sc Assay for Transp osase‐Accessible Chromatin‐seq	*Toxoplasma gondii* from hTERT^x)^ cells	1) The dominant burst in G1 is primarily driven by the transcription factor AP2XII‐8. This factor can promote the expression of 44 ribosomal proteins encoding regulon.	[400]
*Trichuris muris* infections	ScRNA‐seq	Mouse CD236+CD45− DAPI^y)^‐epithelial cells	1) Larvae degrade mucus layers to enter epithelial cells and they are completely intracellular in early syncytial tunnels. 2) The infection progression leads to cell damage and an increase in enterocytes expressing Isg^z)^15, potentially triggering the immune response against the whipworm and promoting tissue repair.	[401]
*Trypanosoma brucei* infections	ScRNA‐seq	Mouse brain CD45+CD11b+ myeloid cells	1) Microglia are enriched around the ventricles, and epiplexus cells are expanded. 2) Resident macrophages enhance parasite defense and thus promote monocyte infiltration across brain barriers. Recruited monocyte‐derived macrophages are more than resident macrophages, which exhibit enhanced transcriptional plasticity and express antimicrobial genes. 3) Border‐associated macrophages show persistent transcriptome alterations, but microglia do not.	[402]
	ScRNA‐seq and spatial transcriptomics	Mouse hypothalamus and coronal brain slices	1) Infection‐induced glial responses are prominently observed near the circumventricular organs, including the lateral and third ventricle. 2) There is an unexcepted interaction between homeostatic microglia and Cd138+ plasma cells, induced by IL^aa)^‐10 and BAFF^ab)^ signaling.	[403]
*Schistosoma* infections	ScRNA‐seq	Juveniles’ stem cells and mother sporocysts stem cells	1) Totally 15 different gene co‐expression modules are identified. Each parasite life‐cycle stage has at least one highly related gene co‐expression module, including numerous long non‐coding RNAs and mRNAs.	[404]
	ScRNA‐seq	*Schistosoma mansoni* miracidia	1) The somatic cells of the larvae account for 93% and the stem cells account for 7%. 2) Each of these stem clusters has sex‐biased transcriptional characteristics, indicating that there is cell type‐specific gene dose compensation for sex chromosome linkage sites.	[405]
	ScRNA‐seq	Mouse hepatic natural killer cells	1) The hepatic natural killer cells can be divided into the mature, immature, memory‐like, and regulatory‐like types. 2) A Thy1+ natural killer cell subset has a strong killing function.	[406]
	ScRNA‐seq	Mouse liver tissues	1) The activation of glucocorticoid receptor reduces the expression of two major transcription factors, Gata3 and Pparg. These two factors increase in infected mouse livers and Th^ac)^2 cells, while decrease after dexamethasone treatment.	[407]
	ScRNA‐seq	Oocytes of *Schistosoma mansoni*	1) There are four cell subsets identified, including somatic, germ cells and progeny, intermediate and late germ cells. 2) SmRAR^ad)^ and associated genes like Smmeiob have the pairing‐dependent feature and ovary‐preferential expression pattern. These genes play the decisive role in oocyte differentiation.	[408]
	ScRNA‐seq	Mouse Peyer's patches	1) In intestinal granuloma, neutrophils, macrophages, T cells and B cells are distributed in layers, and there is obvious aggregation of neutrophils. 2) The egg deposition drives B cell apoptosis, T cell exhaustion, and activation of fibrotic pathways in myeloid cells, which can impair the function of Peyer's patches.	[409]

^a)^ single‐cell;

^b)^ single‐cell RNA sequencing;

^c)^ single‐cell T cell receptor sequencing;

^d)^ interferon;

^e)^ mammalian target of rapamycin;

^f)^ eukaryotic Initiation Factor 2;

^g)^ ribosomal protein small;

^h)^ ribosomal protein large;

^i)^ single‐cell DNA sequencing;

^j)^ SRY‐related high‐mobility‐group box 4;

^k)^ T cell receptor;

^l)^ variable, diversity and joining;

^m)^ myelocytomatosis viral oncogene homolog;

^n)^ nuclear factor kappaB;

^o)^ BCL2 associated athanogene‐1;

^p)^ surface antigen 1;

^q)^ bradyzoite formation deficient;

^r)^ bone marrow‐derived dendritic cells;

^s)^ granule protein;

^t)^ protein targeting to glycogen;

^u)^ long distance migration;

^v)^ chemokine ligand 2;

^w)^ C‐X‐C motif chemokine ligand 5;

^x)^ human telomerase reverse transcriptase;

^y)^ 4,6‐diamidino‐2‐phenylindole dihydrochloride;

^z)^ interferon‐stimulated gene;

^aa)^ interleukin;

^ab)^ B cell activating factor;

^ac)^ T helper cell;

^ad)^ retinoic acid receptor family.

#### 3.6.1 Malaria

Malaria caused by *Plasmodium* is one of the three deadliest infectious diseases, and there are an estimated 249 million cases in 2022.^[^ ^410^ ^]^ *Plasmodium* harbors high plasticity during its life cycle, and its complex life cycle drives the development and transmission of malaria. This cycle starts with mosquito bites, through which sporozoites (the stage transmitted by mosquitoes to humans) enter the host. Sporozoites quickly enter the hepatocytes and then replicate. In the liver, numerous merozoites (the stage of invasion into erythrocytes) are produced, and these mature merozoites enter the bloodstream and invade erythrocytes. And these parasites replicate asexually in erythrocytes, which then rupture, leading to the release of more merozoites that invade new erythrocytes for the next cycle.^[^ ^411^ ^]^ While in mosquitoes, parasites employ a different developmental strategy, including replicative, invasive, and sexual stages.^[^ ^412^ ^]^ The above processes involve in a number of *Plasmodium‐*host interactions (e.g., parasite‐induced surface modification of infected erythrocytes and macrophage ingestion of merozoites). This evidence indicates that understanding malaria requires exploration of a range of responses in both spatial (infection sites or cells) and temporal aspects (different stages of the lifecycle), and therefore single‐cell sequencing technologies are widely used in this field.

*Plasmodium* can be divided into various species, of which the species that infect humans include *Plasmodium falciparum*, *Plasmodium vivax*, *Plasmodium malariae*, *Plasmodium knowlesi*, and *Plasmodium ovale*. Infections caused by these species are particularly emphasized in the following part.

Malaria tropica: *Plasmodium falciparum* can cause the most lethal malaria, known as malaria tropica, and serious complications including organ failure, anemia, and death. A special aspect of *Plasmodium falciparum* infection is its ability to mediate the production of adhesive phenotypes of infected erythrocytes.

1) Changes in *Plasmodium falciparum*

The parasite itself undergoes certain changes, including disrupted development rhythm. Rawat M et al.^[^ ^413^ ^]^ find that under temperature stress, *Plasmodium falciparum* downregulates the global transcription level, while enhancing transcriptional heterogeneity, including upregulated stress‐responsive and regulators of gametocytogenesis genes. This study depicts the dynamic changes of *Plasmodium falciparum* at different temperatures, yielding insights into the mechanism of malaria‐related periodic fever. Florini F et al.^[^ ^414^ ^]^ report that *Plasmodium falciparum* can flexibly regulate the expression of the Var gene, which is a major virulence factor encoding gene. For example, it can enter a state of low expression or even no expression of the var gene, thereby evading the antibody recognition of infected cells.

2) Heterogeneity of host cell response to *Plasmodium falciparum*

As parasites mature within erythrocytes, they can reshape the membrane architecture of infected erythrocytes, leading to alterations in cell rheological properties, such as adhesiveness. The generation of adherent phenotypes is considered a key factor in the development of malaria tropica.^[^ ^415^ ^]^ Apart from erythrocytes, host immune cells also exhibit evident alterations. To be specific, the host upregulates the abundance of atypical B cells in an IFN‐γ‐dependent manner. This cluster can be further divided into IgD+IgM^lo^, IgD‐IgG+ and IgD+IgM+ subclusters, and the first two have a high affinity threshold of antigen activation, indicative of their contribution to limited autoimmune responses to low‐affinity self‐antigens during chronic malaria.^[^ ^416^ ^]^ For NK, NKT and MAIT cells, they respond differently to different doses of *Plasmodium falciparum* sporozoite stimulus. It is worth noting that MAIT cells undergo significant expansion, which may be mediated by mechanisms similar to homeostatic expansion. Additionally, MAIT cells maintain their functionality, indicating their ability to effectively suppress signals that impair their function.^[^ ^417^ ^]^ This underscores the potential value of MAIT cells in countering this infection.

3) Interactions between *Plasmodium falciparum* and hosts

The above findings characterize the changes from the perspective of parasites or the host, whereas Yang ASP et al.^[^ ^418^ ^]^ and Subudhi AK et al.^[^ ^419^ ^]^ capture the changes in both and effectively integrate these messages to identify powerful targets. For instance, *Plasmodium* specific AP2 transcription factor (PfAP2‐MRP) can manipulate the trophozoite development and host cell reprogramming by binding to relevant gene promoters, and subsequently regulate antigenic variation and pathogenicity in a similar manner.^[^ ^419^ ^]^ Clearly, this parasite molecule can serve as a bridge between host‐*Plasmodium falciparum* interactions and a potential therapeutic target of malaria tropica. Besides, single‐cell technologies are also applied to clarify the mode of malaria transmission^[^ ^420^ ^]^ and capture a gene panel to distinguish parasites with different stages.^[^ ^421^ ^]^

Malaria tertiana: *Plasmodium vivax*, the pathogen of malaria tertiana, is the most widely distributed *Plasmodium* that causes human malaria. They can produce dormant liver forms called hypnozoites, which can activate at indefinite times, causing repeated blood‐stage infections.

**(1) Interactions between *Plasmodium vivax* and hosts**

By simultaneously profiling host‐and parasite‐transcriptomes, Ruberto AA et al.^[^ ^422^ ^]^ capture differences between schizonts and hypnozoites at the single‐cell level (e.g., elevated LISP2 expression in schizonts) and their respective impact on hepatocytes (e.g., schizonts inducing more pronounced host transcriptional changes), providing dormancy‐related factors and underlying targets of *Plasmodium vivax* liver‐stage infection. Similarly, Mancio‐Silva L et al.^[^ ^53^ ^]^ also, provide detailed insights into the host‐parasite interaction by integrating micropatterned co‐cultures with scRNA‐seq. They find that hypnozoites maintain their dormant state through transcriptional/translational repression mechanisms and rely on proteolysis to support their viability. Additionally, downregulation of IFN‐responsive genes (e.g., IRF7 and IFI6) in infected cells suggests the parasite's immune evasion mechanism, while also highlighting the role of IFN‐related genes in anti‐parasite. Such interaction occurs not only at the transcriptional level but also at the genetic level. The host mutation and selection can also generate an identified impact on *Plasmodium vivax* evolution.^[^ ^423^ ^]^

## 4 Perspectives

The key molecules provided by bulk detection technologies are already revolutionizing clinical diagnosis and treatment. However, some adverse events (false‐negative or ‐positive cases, variable treatment efficacy, poor long‐term prognosis, etc.) brought by individual and intercellular heterogeneity also challenge the further application of these diagnostic biomarkers or treatment targets.^[^ ^424^ ^]^ Single‐cell sequencing technology, by revealing such heterogeneity (e.g., transcriptional differences between infected and uninfected cells) sheds light on previously unknown aspects of pathogenesis and opening avenues for innovative diagnostic approaches or therapeutic interventions. Nowadays, based on single‐cell sequencing, researchers typically provide single‐cell atlases including changes in cellular abundance, gene expression, cell‐cell interactions, etc., and/or disease‐causing molecular pathways discovered through the integration of in vivo and in vitro experiments. Based on such results, some molecules identified as key factors during infection have the potential to serve as high‐performance diagnostic/predictive biomarkers or therapeutic targets, and their application scopes (specific patient populations, sample types, and cell types) are also identified. For example, it is not the entire population of NK cells or T cells that is recognized as a TB diagnostic biomarker. Single‐cell sequencing technology allows researchers to further subdivide these cell populations and accurately identify subpopulations that can indicate different TB states, subtypes, and lesion extent. Another example concerns the treatment of malaria. Existing antimalarial drugs are mainly artemisinin‐based combination therapy drugs, while partial resistance to artemisinin has become a threat to global malaria control. ScRNA‐seq data indicate the factors driving the dormancy of *Plasmodium vivax* by comparing the effects of schizonts and hypnozoites on host cells, providing targets for therapeutic strategies to disrupt *plasmodium* dormancy. Of note, there are some challenges that need to be overcome before the targets provided by single‐cell sequencing technology can be effectively utilized in clinical settings. The first challenge is the clinical detection of the targets. The targets identified by single‐cell sequencing technology are typically derived from specific cell populations, indicating that clinicians need to isolate cells before conducting target detection. This process requires certain sample volumes and freshness, and thus it introduces considerations regarding the requirement of detection and storage equipment, as well as the cost. Second, single‐cell sequencing data are massive, and how to rapidly process it and then provide results to clinicians in a concise and readable format needs to be considered. Thirdly, revealing the underlying heterogeneity and enhancing the interpretability of diverse disease phenotypes are a great advantage of single‐cell sequencing technology. However, we should also recognize the importance of balancing disease heterogeneity and commonality. Excessive fine partitioning may lead to issues in result reproducibility and target applicability, which will be more prominent in clinical applications. Building upon heterogeneity, summarizing the underlying commonalities of infectious diseases may contribute to the discovery of broad‐spectrum therapeutic targets. Based on literatures included in this work, many of them (HIV infection/AIDS, TB, hepatitis C, bacterial sepsis, etc.) report infection‐related cell exhaustion, suggesting the promise of immunotherapy for infectious diseases. Additionally, the IFN signaling pathway is shared by many pathogens (HIV, herpesvirus, IAV, MTB, *S. aureus*, *Toxoplasma gondii*, *Plasmodium*, etc.) interacting with their hosts. In detail, IFN signaling pathway involves multiple molecules, including the IFN family, IRF family, ISG family, pattern recognition receptor‐related genes and others. In the infected state, the host enhances its ability to combat pathogens such as HIV and *Trichuris muris* by upregulating the expression of some IFN‐related molecules (e.g., IFN‐α and ISG15), which improve CD8+ T cell cytotoxicity, reverse T cell exhaustion, and activate host immune responses. And deleterious variants in the above‐mentioned IFN pathway‐related molecules (such as IRF7) can affect the host's ability to clear pathogens, increasing the host's susceptibility to the pathogen. It can be seen from this that enhancing the IFN signaling pathway is the key for the host to achieve an effective anti‐infection response.

The application of single‐cell sequencing technology in infectious diseases still requires further expansion. One aspect is that the advanced development of eukaryotic single‐cell sequencing technologies leads to the host‐biased analysis of infectious diseases. It is well known that the intricate properties of infectious diseases (pathogen, host, and their interaction) exponentially increase their complexity, requiring investigators to study alterations on each side, as well as the interplay between them, to understand the overall outcome. Although some prokaryotic or dual scRNA‐seq methods have been reported, these methods have only been applied in some specific infections, and their applicability and performance to other infectious diseases remain to be verified. It is noted that sequencing sensitivity is mainly affected by the abundance of genetic materials and is limited, and therefore, the sensitivity limitation is more prominent in prokaryotic or dual single‐cell sequencing. Recent methodological advancements have emerged to address the above limitations, including implementation of positive‐selection enrichment strategies for selective depletion of structural RNAs from all RNA, dual‐library sequencing enrichment through solution hybridization selection with bacterial transcriptome‐specific probes, improvement of sequencing depth, and so on. Based on such advancements, PatH‐Cap, VASA‐seq, and some other novel methods have been proposed to achieve high‐sensitivity sequencing and analysis of low‐abundance transcripts.^[^ ^425^, ^426^ ^]^ While their applicability for prokaryotic or dual single‐cell sequencing remains to be verified.

The second aspect is that progress in priority areas like drug resistance has been extremely limited, which may be due to the inherent complexity of drug resistance in infectious diseases. Drug resistance can be classified as primary and acquired resistance, and both of these categories can further be divided as mono‐, multidrug‐, and extensive drug‐resistance. If the above‐mentioned types of drug resistance are not distinguished, it can lead to excessive heterogeneity in single‐cell sequencing data. However, the identification of drug resistance subtypes presents difficulties. Discordance between molecular and phenotypic drug‐susceptibility results is common, posing a challenge for accurate assessment. Furthermore, effectively distinguishing between cells containing drug‐resistant strains and those that do not, as well as determining the type of resistant strain present within cells, remains a significant difficulty. Fortunately, the advancement of prokaryotic and dual single‐cell sequencing technologies has brought a glimmer of hope for addressing such complex issues.^[^ ^29^ ^]^ From a basic science perspective, these technologies can analyze the heterogeneity among bacterial cells and the influence of host immune status on bacterial activities (e.g., the expression of drug resistance‐related genes in different cells), which may explain this inconsistency to some extent. From a clinical perspective, we can integrate phenotypic drug‐susceptibility results and single‐cell gene profiles to construct drug resistance‐specific gene references. By comparing the similarity of gene profiles between the sample and the reference, resistant bacteria and corresponding infected cells could be precisely inferred or even identified. This way can improve the accuracy of molecular diagnosis to some extent. However, these emerging technologies are still in their infancy and require further validation, especially when facing challenges such as low bacterial abundance and detecting the target in different sample types.

ScRNA‐seq and single‐cell immune repertoire sequencing have been extensively applied in infectious diseases; the future holds great potential with the application of emerging techniques (full‐length total RNA single‐cell sequencing, single‐cell high‐throughput chromosome conformation capture sequencing, spatial transcriptomics, etc.).^[^ ^427^, ^428^ ^]^ For instance, Hildebrandt F et al.^[^ ^429^ ^]^ integrate spatial and single‐cell transcriptomics to reveal a detailed spatio‐temporal landscape of host‐*Plasmodium* interactions in the liver, including changes in lipid metabolism near parasite infection sites, different inflammatory programs among lobular regions, and areas where different inflammatory cells are enriched. Such detection can elucidate the behavior of pathogens and host cells in various tissues from a three‐dimensional perspective, providing a more realistic biological profile. Single‐cell sequencing technology based on third‐generation sequencing has been developed, and it provides a unique perspective for the analysis of chronic lymphocytic leukemia relapse,^[^ ^430^ ^]^ suggesting that the application of this emerging technology in infectious diseases may lead to new discoveries. Moreover, current research in the field of infectious diseases uses single‐cell sequencing to detect whole‐genome information. Single‐cell targeted sequencing should be focused on due to its advantage of removing redundant data, deepening sequencing depth, and reducing the cost. These advancements can pave the way for robust prevention, precise diagnosis and personalized treatment of infectious diseases, as well as improving our ability to respond to infectious disease outbreaks.

## 5 Conclusion

This review synthesized cutting‐edge methodological progress in single‐cell sequencing technologies applied to infectious disease research, with particular emphasis on transformative advancements in prokaryotic and dual single‐cell sequencing. These technological breakthroughs collectively provide unprecedented resolution for understanding pathogenesis from different perspectives (pathogens, host, and their interactions), identifying diagnostic biomarkers and therapeutic targets, and ultimately advancing precision medicine. Despite significant heterogeneity observed among diseases, individuals, or cells, alterations in the IFN‐related pathways and cell exhaustion appear to be shared characteristics in many infectious diseases, which is important to develop broad‐spectrum therapeutic strategies. Additionally, the application of single‐cell technologies in infectious diseases can be further expanded, such as the exploration of drug resistance, pathogen behaviors, and host‐pathogen interactions. These efforts are of great importance in reducing the burden of infectious diseases.

## Conflict of Interest

The authors declare no conflict of interest

## Author Contributions

M.L., Y.L., and J.Z. contributed equally to this work. M.L., J.Z., H.L., J.C., and B.Y. designed this work and acquired funding support. M.L., Y.L., J.Z., H.L., H.R., D.W., S.Z., and X.Z. collected relevant papers. W.M., Y.H., and S.L plotted figures. All authors wrote, reviewed, and edited this manuscript.

## Supporting information



Supporting Information
