# Supplementary material for: Advances in Single‐Cell Sequencing for Infectious Diseases: Progress and Perspectives
Source: Adv Sci (Weinh). 2025 Jul 4;12(32):e15678. doi: 10.1002/advs.202415678 (PMC12407387; doi:10.1002/advs.202415678)
Supplement: Supplementary file 1 — Supporting Information [file ADVS-12-e15678-s001.docx]

**Advances in Single-Cell Sequencing for Infectious Diseases: Progress and Perspectives**

*Mengyuan Lyu^#^, Yang Liu^#^, Jian Zhou^#^, Hongli Lai, Hongxia Ruan, Dongsheng Wu,*

*Shun Zhu, Xudong Zhou, Wananqi Ma, Yuchen Huang, Shuting Lei, Han Luo^*^, Jie Chen^*^, Binwu Ying^*^*

* Corresponding author

H. Luo: [luohan-hx@scu.edu.cn](mailto:luohan-hx@scu.edu.cn)

Jie Chen: chenjiewch@wchscu.cn

Binwu Ying: [yingbinwu@scu.edu.cn](mailto:yingbinwu@scu.edu.cn)

This Supporting Information file includes:

1. **Supplementary Table 1.** Potential therapeutic targets for infectious diseases.
2. **Supplementary Figure 1.** Stacked bar chart showing proportion of included individuals in the discovery cohort.

**Supplementary Table 1** Potential therapeutic targets for infectious diseases^*^.

| **Category** | **Targets** | **Some examples of applicable infections or diseases** |
| --- | --- | --- |
| IFN ^a)^ signaling- related targets | IFN-I ^**^ | HIV^b)^ infection/AIDS^c)^ and *Salmonella* infection |
|  | IFN-α | HIV infection/AIDS and hepatitis B |
|  | IFN-γ | Sepsis and malaria tropica |
|  | IRF7^d)^ | HSV-1^e)^ infection and malaria tertiana |
|  | IL^f)^-1α | *Staphylococcus aureus*-related cutaneous inflammation |
|  | IL-1β | Sepsis, *Staphylococcus aureus*-related cutaneous inflammation and TB^g)^ |
|  | IL-4Rα | *Staphylococcus aureus*-related atopic dermatitis |
|  | IL-6 | TB |
|  | IL-10 | *Trypanosoma brucei* infection |
|  | IL-10R and IL-15 | HIV infection/AIDS |
|  | IL-17A | *Staphylococcus aureus*-induced sepsis |
|  | ISGs^h)^ | HCMV^i)^ infection, sepsis and *Trypanosoma brucei* infection |
|  | IFI6^j)^ | Malaria tertiana |
|  | TLR^k)^2 | *Cutibacterium acnes*-induced acne |
|  | TLR4 | *Escherichia coli* induced-liver abscesses |
|  | TLR7 | HIV infection/AIDS |
| Cell exhaustion-related targets | KLRG1^l)^, PD-1^m)^, CTLA4^n)^, CD^o)^160, TIM-3^p)^, CMC1^q)^, SH2D1A^r)^, COMMD6^s)^, TRAPPC1^t)^, COTL-1^u)^, IRF^v)^1, ITGB1^w)^, GZMB^x)^, PRF1^y)^, GNLY^z)^, TMSB4X^aa)^, PFN1^ab)^, ACTG1^ac)^, ARPC1B^ad)^, TRAPPC1^ae)^ and T-bet^dim^Eome^shi af)^ | HIV infection/AIDS |
|  | LAG3^ag)^, LAYN^ah)^, TOX^ai)^, HAVCR2^aj)^, LAG3, PDCD1^ak)^ and TIGIT^al)^ | Hepatitis B |
|  | KLRG1 and TIGIT | Epstein-Barr virus infection |
|  | TIGIT, LAG3, PDCD1, CTLA-4, LAG3, HAVCR2 and PVR^am)^ | Lepromatous leprosy |
|  | H1FX^an)^, ZFP36^ao)^, VIM^ap)^, PPP1R15A^aq)^, ITM2C^ar)^, HLA-DRA^as)^, PDCD1 and TNFRSF^at)^9. | TB |
|  | TNFSF10, TNFRSF1B, BCL2L11^au)^, CASP3^av)^, PRDM1^aw)^, LAG3, SPl1^ax)^, IRAK1^ay)^, STAT^az)^3, SMAD3^ba)^, PRTN3^bb)^, IL1B, PRMT4^bc)^ and LPS^bd)^, | Sepsis |
|  | TOX1, CD39, CD38, PD-1, CD137 and IRF4 | Hepatitis C |
|  | EOMES, TB^be)^X21, PDCD1, ZEB2^bf)^, CD160, KLRE1^bg)^, CXCR^bh)^6, CXCR1, IL21R, BATF^bi)^, IL-18R, IRF7, STAT1, STAT2, [CXCL^bj)^10](https://www.sciencedirect.com/topics/immunology-and-microbiology/gamma-interferon-inducible-protein-10), HAVCR2, LAG3, CTLA4, CD160 and CD244 | LCMV^bk)^ infection |

* This table listed some main targets that were related to IFN-signaling pathways and cell exhaustion.

** The failure to obtain was to take a specific member of the gene family.

^a)^interferon; ^b)^human immunodeficiency virus; ^c)^acquired immune deficiency syndrome; ^d)^interferon regulatory factor 7; ^e)^herpes simplex virus types 1; ^f)^interleukin; ^g)^tuberculosis; ^h)^ interferon-stimulated genes; ^i)^human cytomegalovirus; ^j)^interferon alpha inducible protein 6; ^k)^Toll-like receptor; ^l)^killer cell lectin-like receptor subfamily G member 1; ^m)^programmed death-ligand; ^n)^cytotoxic T lymphocyte-associated antigen 4; ^o)^cluster of differentiation; ^p)^T cell immunoglobulin and mucin domain-containing protein 3; ^q)^C-X9-C motif containing 1; ^r)^SH2 domain containing 1A; ^s)^copper metabolism MURR1 domain; ^t)^trafficking protein particle complex subunit 1; ^u)^coactosin-like protein; ^v)^interferon regulatory factor 1; ^w)^integrin beta1; ^x)^Granzyme B; ^y)^Perforin; ^z)^granulysin; ^aa)^Thymosin beta 4; ^ab)^profilin 1; ^ac)^gamma-actin; ^ad)^actin-related protein 2/3 complex subunit 1B; ^ae)^trafficking protein particle complex subunit 1; ^af)^eomesodermin; ^ag)^lymphocyte-activation gene 3; ^ah)^layilin; ^ai)^thymocyte selection-associated high mobility group box protein; ^aj)^hepatitis A virus cellular receptor 2; ^ak)^programmed cell death 1; ^al)^tyrosine-based inhibitory motif domain; ^am)^poliovirus receptor; ^an)^Histone 1FX; ^ao)^the zinc finger protein 36; ^ap)^vimentin; ^aq)^protein phosphatase 1 regulatory subunit 15A; ^ar)^integral Membrane Protein 2C; ^as)^human leukocyte antigen-DR alpha; ^at)^the TNF Family of Ligands and Receptors; ^au)^B cell lymphoma 2 like protein 11; ^av)^caspase-3; ^aw)^PR domain zinc finger protein 1; ^ax)^squamosa promoter binding protein-like gene 1; ^ay)^interleukin-1 receptor associated kinase 1; ^az)^signal transducer of activation; ^ba)^suppressor of mother against decapentaplegic 3; ^bb)^proteinase 3; ^bc)^ protein arginine methyltransferase 4; ^bd)^lipopolysaccharide; ^be)^T-bet; ^bf)^zinc finger E-box binding homeobox 2; ^bj)^killer cell lectin-like receptor subfamily E1; ^bh)^C-X-C motif receptor; ^bi)^basic leucine zipper ATF-like transcription factor; ^bj)^C-X-C motif ligand; ^bk)^lymphocytic choriomeningitis virus.


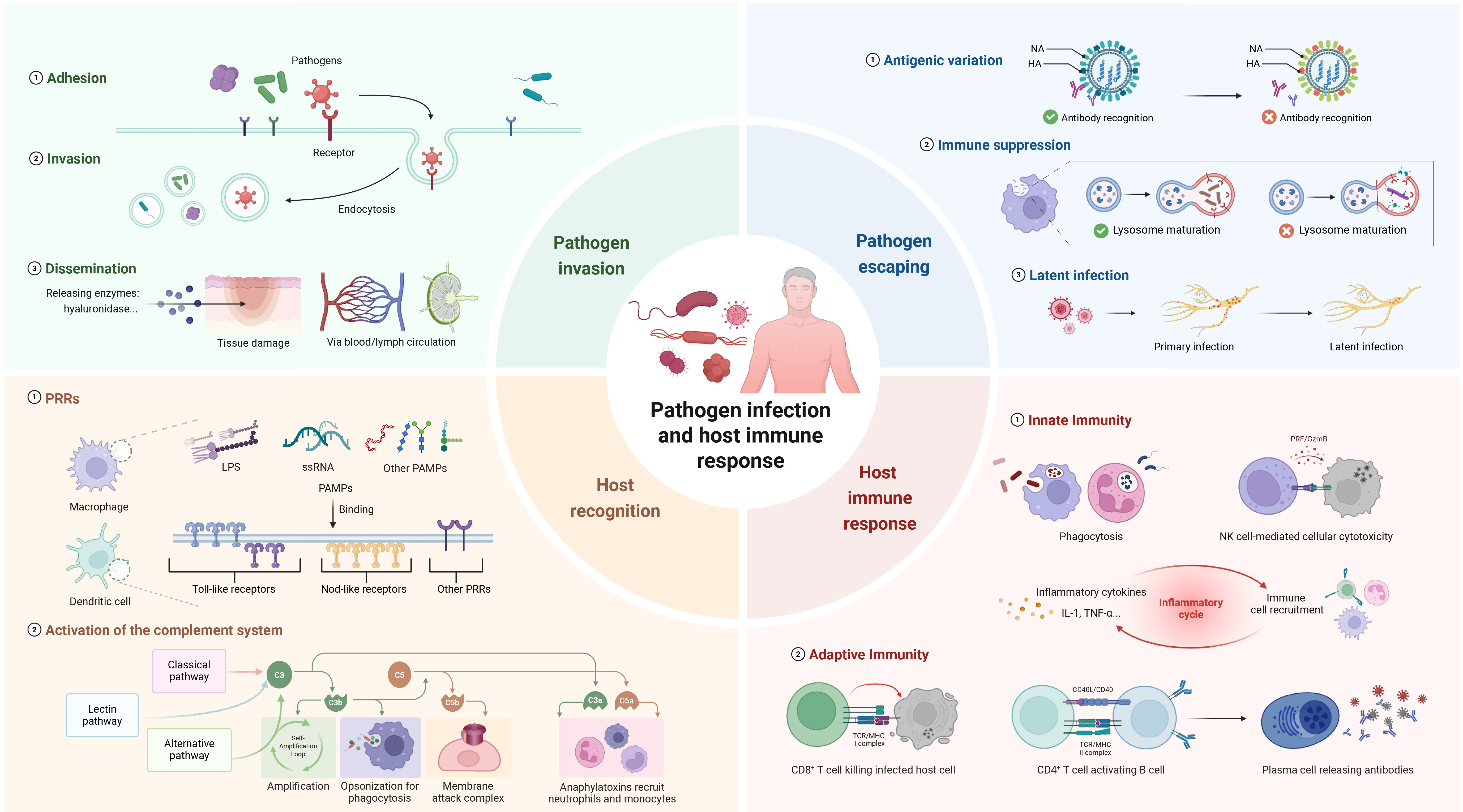


**Supplementary figure 1 Mechanism diagram of pathogen and host interactions.**

PRRs: pattern recognition receptors; LPS: ‌lipopolysaccharide; ssRNA: single-stranded RNA; PAMPs: ‌pathogen-associated molecular patterns; IL-1: interleukin-1; TNF-α: tumor necrosis factor-alpha; TCR: T Cell Receptor; MHC: major histocompatibility complex; NK: natural killer; NA: neuraminidase; HA: hemagglutinin
